# Supplementary material for: Ancient human DNA recovered from a Palaeolithic pendant
Source: Nature. 2023 May 3;618(7964):328–32. doi: 10.1038/s41586-023-06035-2 (PMC10247382; doi:10.1038/s41586-023-06035-2)
Supplement: Supplementary file 1 — Supplementary Information [file 41586_2023_6035_MOESM1_ESM.pdf]

nature portfolio

https://doi.org/10.1038/s41586-023-06035-2

Supplementary information

Ancient human DNA recovered from a Palaeolithic pendant

In the format provided by the authors and unedited

Title: Ancient human DNA recovered from a Paleolithic pendant

Authors: Elena Essel[1*†], Elena I. Zavala[1,2,3*], Ellen Schulz-Kornas[4*], Maxim B. Kozlikin[5], Helen Fewlass[1], Benjamin Vernot[1], Michael V. Shunkov[5], Anatoly P. Derevianko[5], Katerina Douka[6], Ian Barnes[7], Marie-Cécile Soulier[8], Anna Schmidt[1], Merlin Szymanski[1], Tsenka Tsanova[9], Nikolay Sirakov[10], Elena Endarova[11], Shannon McPherron[1], Jean-Jacques Hublin[1,12], Janet Kelso[1], Svante Pääbo[1], Mateja Hajdinjak[1,13], Marie Soressi[14*†], Matthias Meyer[1*†]

* These authors contributed equally to this work
† Corresponding authors

Correspondence to: elena_essel@eva.mpg.de, m.a.soressi@arch.leidenuniv.nl, mmeyer@eva.mpg.de

Contents

SI1: Sample provenance, sample information and archaeological context 2
Faunal remains used for testing reagents 2
Modified bone and tooth specimens used for non-destructive DNA extraction 2
Three freshly excavated pendants from Bacho Kiro Cave 3
A freshly excavated pendant from Denisova Cave 4
SI2: Testing reagents for non-destructive DNA extraction using 3D surface texture measurements 10
Background 10
3D surface texture (3DST) analysis 11
SI3: Compatibility of the method with radiocarbon dating 18
SI4: Taxonomic assignment of mtDNA sequences 21
SI5: Denisova Cave pendant – human mtDNA analysis 23
Patterns of cytosine deamination and estimates of present-day human contamination 23
Number of mtDNA haplotypes and consensus calling 24
Tree building and molecular branch shortening 26
SI6: Denisova Cave pendant – cervid mtDNA analysis 33
Consensus calling 33
Generating additional ancient wapiti mtDNA genomes 34
Tree building and genetic dating 35
SI7: Denisova Cave pendant – human nuclear DNA analysis 42
Nuclear DNA processing and contamination estimates 42
Data filtering for population genetic analyses 43
Principal Component Analysis (PCA) 43
f3-statistics 44
D-Statistics 44
Sex determination 45
References 57

SI1: Sample provenance, sample information and archaeological context

Faunal remains used for testing reagents

Ten unmodified faunal remains were selected within the faunal collection excavated at two limestone cave sites located in the center-west of France: Quinçay and Les Cottés. Archaeological finds and features are preserved in a sandy-silty matrix at Quinçay[1] and a sandy-clayish matrix at Les Cottés[2]. At Les Cottés, large quantities of centimeter to decimeter size limestone clasts as well as faunal remains are present all along the stratigraphic sequence, meanwhile at Quinçay they are only present in the upper part of the stratigraphy (layer Em and Ej). Both cave sites contain only late Middle Palaeolithic and early Upper Palaeolithic deposits[3-5]. Les Cottés archaeological deposits have been radiometrically dated to circa 55-35 thousand years ago (ka)[2,6]. The Quinçay sequence has not been dated radiometrically yet. Considering the cultural and ecological similarities with other Châtelperronian sites[3], it is expected that Quinçay faunal remains are 45-35 ka[4].

Ten unmodified bone fragments and teeth were selected for testing reagents (seven from Quinçay and three from Les Cottés; Extended data table 1, Extended data fig. 2) because of their similarity in size and shape to material typically used for osseous artefact production at these two sites[7]. The seven Quinçay faunal remains were excavated by F. Lévêque between 1971 and 1980 and all but one was uncovered in the Châtelperronian layers Em and Ej. Considering that only Châtelperronian layers preserve Pleistocene fauna at Quinçay[4], it is likely that the Quinçay specimen uncovered in reworked sediment originates from a Châtelperronian context. The Les Cottés specimens selected for testing reagents were excavated in 2018 by M. Soressi and her team in reworked sediment. As the late Middle Palaeolithic and early Upper Palaeolithic layers are the only archaeological layers preserved at Les Cottés, it is likely that these three specimens originate from one of these layers and date to the 55-35 ka time window.

Modified bone and tooth specimens used for non-destructive DNA extraction

A total of 15 osseous specimens were used for non-destructive DNA extraction. Eleven specimens were excavated 50 to 32 years ago from the Châtelperronian layers of

Quinçay (likely dating to 45-35 ka[3,4]) by F. Lévêque: four modified bones, one modified and one weathered ivory fragment, and five other specimens that are preserved too poorly to determine whether they were modified or used prior to further microscopic analysis (Extended data table 1, Extended data fig. 3). Similar to the specimens used for testing reagents (see above), they originate from a sandy-silty matrix with small quantities of centimeter to decimeter size limestone clast, except for the ones coming from layer Ej where clast can be much more abundant[1]. They were excavated and curated without wearing gloves. Some of them had been washed with water, labelled with ink and likely consolidated using glue following common practice at the time of excavation.

Three freshly excavated pendants from Bacho Kiro Cave

Three teeth pendants (Extended data table 1, Extended data fig. 3) were recently excavated at Bacho Kiro Cave, Bulgaria[8-10]. They were unearthed in 2018 (pendant CC8-1571, (BKP3)) and in 2019 (pendants DD7-218 (BKP1) and DD8-187 (BKP2)) in the excavated area Niche 1, from layers belonging to the Initial Upper Palaeolithic (IUP). The specimens were excavated while wearing gloves and using wooden spatulas. All pendants were excavated together with the surrounding sediment and stored in cool conditions until they were sampled.

Pendant DD7-218 (BKP1) was excavated in the upper part of main diagnostic IUP Layer I, which consists of dark brown loamy clay and accumulated due to anthropogenic activities. The tooth, which belonged to an unidentified mammal, is covered by a white patina and the surface is rounded and smoothed.

Pendant DD8-187 (BKP2), an ursid right second molar, was excavated in a slightly different context - the contact zone with the upper layer, called Layer H/I, which is characterized by a sharp shift in sedimentation mode and erosional processes[8]. The pendant's surface is eroded and patinated, which together with its smoothed root surface and broken perforation suggest that it was altered by post-depositional movements. In addition, it carries a crack along its surface.

Pendant CC8-1571 (BKP3), an ursid right second incisor, was excavated in the contact zone with the lower layer, called Layer N1- I/J, which is a greenish brown loamy clay of

endokarstic origin containing limestone fragments. The tooth root is eroded and the pendant cracked and patinated.

Radiocarbon dates for the contact zones of Layer N1-I/J and Layer N1-I to the upper part of Layer N1-I (obtained from faunal bones mostly with anthropogenic modifications and four H. sapiens remains) range from 45,040 to 43,280 years cal B.P. (95.4% probability; radiocarbon dates reported in Fewlass et al. (2020)[11] were recalibrated using the IntCal20 calibration curve[12] using OxCal version 4.4[13]). The extensive set of C14 dates is consistent with the stratigraphy and together with high artefact densities implies relatively continuous human use of the cave during this interval[11].

A freshly excavated pendant from Denisova Cave

The 2019 excavations undertaken in the middle part of the South Chamber of Denisova Cave have yielded a pendant (DCP1) made from the canine of a deer found in the upper part of layer 11 in square E-3 (Extended data table 1, Extended data fig. 3 and 4). Upon recording the coordinates, the pendant was placed immediately into a sterile plastic bag. All handling was made using sterile gloves and protective masks to prevent contamination of the sample with modern genetic material. After removal from the layer and prior to transferring to the laboratory, the pendant was stored in a refrigerator at 5 °C. The temperature of the surrounding sediments at the time of identification was 6.3 °C. In the coordinate system of South Chamber, square E-3, where the pendant was found, is 23 m from the main entrance to the cave.

Cleaning of the pendant during non-destructive DNA extraction revealed that it is complete and in an excellent state of preservation. Shallow cracks are visible in the root that are likely to have occurred while the object was in use. The surface is generally smooth, without secondary formations, and traces of mineral coloring pigment are present in the pendant hole. The excellent state of preservation is indicative of minimal movement of the object in the ground, which is mostly coarse rubble-grusy aggregate (see below for a detailed description of the sediment). With intensive movement in such a sediment, damage to the ornament would have been inevitable.

Stratigraphic context of DCP1

The pendant was found in the upper part of lithological layer 11 (Extended data fig. 4). The top and base deposits of layer 11 in this area correspond to the levels of -150 and -230 cm, respectively. The actual thickness of layer 11 does not exceed 50 cm. The relatively wide range of depths within the square is because layer 11, like the underlying Pleistocene sediments, dips toward the southwestern rock wall of the chamber at an angle ranging from 20–25 to 50°. Layer 11 appears in the chamber as two stripes extending along the rock walls of the cave. Such deposition, as the preliminary data show, was possible because of post-sedimentary visco-plastic deformations and the characteristics of the bedrock forming the chamber floor. However, the stratigraphic succession of layers has remained intact and no mixing of sediments from different layers took place.

The sediments from layer 11 include light loam incorporating large amounts of clastic limestone. Unmodified clasts were found to be predominantly of small and medium size, revealing sporadic inclusions of coarse fractions that show isometric (cube-shaped) and moderately flattened forms; limestone clasts show sharp edges and tips, demonstrates chaotic orientation and, in places, reveals a poorly developed (less than 1 mm) corrosion rim. The fill material comprises grey-brown, sandy, and scree-reached, light to medium loam, actively reacting with hydrochloric acid (HCl). The inclusions contain well-preserved solid bones showing a light grey surface. The lower boundary of layer 11 is clear and sharp; it can be recognized by the change in color of the fill material. The lower deposits of layer 11 include a 2–12 cm thick horizon of intensely darkened sediments containing the inclusions of finely crumbled charcoal, being visible in all parts of the layer.

Faunal remains

The bone remains from large mammals found in layer 11 are mainly comprised of unidentifiable fragments (~ 90%). The species composition of identifiable fauna has been determined by Prof. S. Vasiliev[14]. Bones from *C. crocuta spelaea*, *Canis lupus*, *Vulpes vulpes* and *Ursus arctos* are the most abundant predators, whereas bones from *Panthera spelaea* are less numerous. Ungulate animals are mainly represented by *Capra sibirica*, *Ovis ammon*, *Bison priscus*, and horses (*Equus. ovodovi / ferus*). Skeletal remains from

Capreolus pygargus, *Cervus elaphus sibiricus*, *Megaloceros giganteus*, *Rangifer tarandus* and *Coelodonta antiquitatis* are also present. The composition of large fauna from layer 11 generally indicates the mosaic pattern of landscapes typical of highlands, with the domination of species from open biotopes.

The collection of bones from small vertebrates identified by Prof. A. Agadjanian[15] includes 3,146 specimens. The number of bats accounts for 2.6%. *Sorex* 0.5%, *Asioskalops* 2.2%, *Spermophilus* 1.1%. The proportion of *Crisetulus barabensis* accounts for 0.3%, *Allocricetulus eversmanni* 1%. The number of *Clethrionomys*, 1%; *Alticola*, 16.5%; *Lagurus*, 8.4%; common *Microtus*, 23.6%; *Arvicola*, 0.2%; *Myospalax myospalax* 3.1%; *Ochotona* 1.3%. Bones identified as belonging to *Lepus* 0.2%; *Mustela* and *Martes*, by 0.3%. The number of bones from birds 19.2%. The proportion of reptile accounts for 0.5%, amphibs 0.1%, and fishes 8.3%. The overall composition of microvertebrates from layer 11 suggests the expansion of open landscapes during its formation.

Radiocarbon chronology

Currently, two radiocarbon age determinations have been produced for the context of the pendant. The ages were obtained on charcoal samples collected in 2018 from square 3-3 (1.5 m away from the pendant) in the middle (OxA-X-3089-11) and lower (OxA-X-3089-12) parts of layer 11 (in the sequence of deposition).

Both charcoal samples were identified by an experienced anthracologist prior to dating; both dated fractions belonged to *Salix/Populus cf*. The charcoals were pretreated using the AOx-SC method (ORAU pers. comm.) and the analytical parameters and new dates are given in Supplementary table 1.1. Both samples yielded between 73-80% C.

The two ages (OxA-X-3089-11: 19,990 ± 61 BP) and (OxA-X-3089-12: 33,500 ±220 BP) calibrate to 24,200–23,830 cal BP and 39,180–37,560 cal BP, respectively, at 95.4% probability using IntCal2020[12] and the OxCal platform (4.4 version)[13]. The difference in the obtained dates and the stratigraphic profile exposed at this part of the South Chamber indicate a relatively large time range for the formation of layer 11 in South Chamber (at least 24-39 ka), which is generally consistent with the biostratigraphic data. In addition, the younger of the radiocarbon dates corresponds well with the molecular genetic age

estimates obtained from DNA extracted from the pendant (see Supplementary information 5 and 6).

Description of DCP1 and its archaeological context

The pendant found in square E-3 of layer 11 was manufactured from the canine of a deer (Extended data fig. 3 and 4). The biconical hole for hanging was made using a double-sided drilling technique in the root section of the tooth, which seems to have been aligned by scraping and grinding before drilling. The hole shows traces from a light red mineral dye with a pinkish tint. Pendants of such a type are one of the most common types of articles for personal adornment in the collections attributed to the Initial and Early Upper Palaeolithic from Denisova Cave[16]. Direct dating of two pendants of this type recovered from layers 11.2 and 11.1 in East Chamber of the cave has yielded ages of 41,900–38,400 years BP (OxA-30005) and 32,660–31,100 years BP (OxA-30006) at 95.4% probability, respectively[17].

Other non-utilitarian objects in the collection from layer 11 of South Chamber include beads, pendants, tubular beads, rings, fragments of tiara and bracelet made from animal teeth and bones, mammoth tusk, soft ornamental stone, such as serpentine, talc, and chrysotile, ostrich eggshells, freshwater mollusk shells – over 50 items in total. Particular attention should be given to a zoomorphic figurine, most likely of a feline predator, missing the head and forelimbs that were lost in antiquity. The statuette is ornamented on all sides with uniform rows of four short notches showing inside the remnants of a red mineral pigment[16]. A marble pebble showing traces of ochre and the fragment of a red mineral pigment revealing signs of scraping should also be regarded as evidence for symbolic behavior. The formal bone tools from layer 11 of South Chamber include needles with a drilled eye, points, polishers, slotted pieces, spatulas, and awls. It is important to note that most of the non-utilitarian objects and bone tools were found in the lower part of layer 11, in the underlying dark, charcoal-saturated horizon.

The primary flaking method recognized in the Early Upper Palaeolithic stone tool industry from layer 11 of South Chamber is characterized by parallel knapping aimed at producing elongated flakes, large blades and small bladelets. Radial flaking was used to produce short and shortened flakes. The tool assemblage includes end-scrapers, burins, and

retouched blades showing a recognizable Upper Palaeolithic morphology. However, this industry has a significant proportion of various types of side-scrapers, denticulate, notched, and spur-like forms. This is more characteristic of the lower and middle parts of the layer, while its upper deposits show a high concentration of small bladelets and microblades, including backed pieces.

Supplementary table 1.1. Summary statistics of radiocarbon dated charcoal samples. Radiocarbon dating results for the two charcoals found in the vicinity of the Denisova pendant.

Sample ID/ Field number	PCode	Material	Genus	Used (mg)	mg C	%C	δ13C	OxA	Date	±
DC-35/ N821	AOx-SC	charcoal	*Salix/ Populus*	34.1	19.674	73.1	-23.5	X-3089-11	19990	61
DC-37/ N827	AOx-SC	charcoal	*Salix/ Populus*	104	27.793	80.6	-26.6	X-3089-12	33500	220

SI2: Testing reagents for non-destructive DNA extraction using 3D surface texture measurements

Background

Artefacts such as ornaments and tools made of animal skeletal remains preserve microtopographical surface characteristics of the material they were made from (material structure), as well as characteristics associated with their use. For instance, the microtopography of bone artefacts preserves characteristics such as rounding and curvature of the bone and microscopically small furrows and plateaus[18,19], features that are consistent with the use of the bone tool on an animal skin[20]. Thus, in addition to preserving visual integrity, a method for non-destructive DNA extraction from artefacts should also preserve the microtopography of the material.

In 2004, Rohland and colleagues presented a non-destructive method for the isolation of DNA from historical tissues (< 200 years old), including bones and teeth[21]. A later study evaluated the method for the use with more ancient material (human teeth and bone fragments from Neolithic and Chalcolithic sites), and reported alterations to the inner structure of the specimens, specifically their porosity[22], indicating that the method does not fully preserve the integrity of skeletal remains older than a few hundred years. Both studies relied exclusively on visual comparison of the material before and after DNA extraction, without examining possible alterations to the microtopography.

Here we use quantitative 3D-surface texture analysis (3DST[23]) to re-evaluate the suitability of the method by Rohland et al. (2004) for DNA extraction from Pleistocene bones (guanidinium thiocyanate (GuSCN) reagent, see Methods section) and to explore alternative strategies for the non-destructive release of DNA from ancient bones and teeth using ethylenediaminetetraacetic acid (EDTA), sodium phosphate and sodium hypochlorite (bleach) as reagents (see Methods section). 3DST allows for the precise measurement of the surface microtopography characteristics in relation to the functional use and handling and artefacts. During the past 10 years, 3DST has been applied as a light-optical and non-destructive method to measure the structure of both tooth as well as bone surfaces (for a review see Schulz-Kornas et al. (2020)[24]). The method has been

adapted for anthropological, biological, and archaeological applications in wear studies on teeth[23,25-29] and lithics[30-35], and less frequently on ochre[36] and bone tools[37-39].

3D surface texture (3DST) analysis

To enable repetitive quantitative 3D surface texture measurements at the exact same location on the sample, we drilled three or four holes to mark the corners of an 800x800 µm surface area that was measured before and after treatments. The holes were circa 150 µm deep and were drilled using a sterile 0.2 mm dentist drill at lowest speed and with as little pressure as possible. Prior to the measurements, we fixed the bone/tooth samples on a grid paper containing the outline of the sample using dissection needles.

We scanned the surface before and after the treatments at multiple loci with a confocal disc-scanning microscope (µsurf mobile, Nanofocus AG, Oberhausen, Germany) using a 20x lens (numerical aperture = 0.4, field of view = 0.8 mm^2). Because of its large working distance (12 mm), the 20x lens has the advantage of covering a large field of view and allowing for large sample curvature (large δz variation). The quality of the surface texture scan was reviewed after each scan was completed and scans with 95% or more valid points of the measured surface were accepted following the established measuring protocol[19,23] . At least four surface scans per sample were collected and analyzed.

We applied metrological pre-processing to each scan to reduce the nominal form by filtering long-scale components of the surface (waviness) from short-scale components (surface roughness and noise) following ISO 25178[40] recommendations for technical surface scans. Here long-scale components are bone/teeth curvature, form, and shape, while short-scale components are wear-traces and measurement noise. A combination of a filter and an operator was used to reduce measurement noise applying a low pass S-Filter and a F-Operator as form removal as applied on bone tools by Martisius et al. (2019, 2020)[18,19]. We used Mountains Map® Premium (version 7.4.8076 analysis software by Digital Surf (Besançon, France) with the following specifications (in brackets): leveling (least square method), outlier removal (outlier removal method: removal of isolated outliers and those around edges, with normal strength, and fill in of holes <225 points, removal of noise), and fill in of non-measured points (smoothing method calculated from neighbors). Each non-measured point is replaced by a value obtained from the

neighboring valid points. The F-operator remove form was set using a polynomial of second order (polynomial of degree 2). From the meshed axiomatic 3D models, we chose the following four of the thirty ISO 25178 parameters for statistical testing (paired t-test, before and after the treatment, $\alpha \leq 0.01$): mean roughness (*Sa*) and void volume (*Vvv*), peak curvature (*Spc*) and peak density (*Spd*).

Microtopographic surface alterations on the bone samples increased significantly with EDTA (Supplementary table 2.1, Extended data fig. 1 and Supplementary fig. 2.1), resulting in increased surface roughness as indicated by increased heights (larger arithmetic mean height, *Sa*), deeper voids (larger Void volume, *Vv*), and more rounded and more frequent peaks (larger peak curvature, *Spc;* higher peak density, *Spd*). In contrast, all four parameters varied less after treatment with phosphate buffer, bleach and water, with only an increase in voids (*Vvv*) being barely statistically significant ($p = 0.008$) for the phosphate treatment.

On the enamel and dentine surfaces of teeth, both EDTA and GuSCN treatment led to significant surface alterations (Supplementary table 2.1, Extended data fig. 1 and Supplementary fig. 2.2), but to a lesser extent than observed for bone samples with EDTA. GuSCN treatment on enamel led to increased surface roughness, as indicated by significantly increased heights (smaller arithmetic mean heights, *Sa*) and peak curvatures (*Spc*), as well as insignificantly larger voids (void volume, *Vv*) and more peaks (peak densities, *Spd*). On dentine, the opposite pattern was observed with GuSCN treatment, *i.e.* decreased roughness as indicated by significantly decreased heights (*Sa*), decreased void volume (*Vv*), more rounded peaks (increased *Spc*) and more frequent peaks (higher *Spd*), with the latter three parameters not reaching significance. EDTA treatment on dentine resulted in increasing roughness as well; as indicated by significantly larger heights (*Sa*) and more rounded peaks (*Spc*). Bleach treatment significantly reduced peak densities (*Spd*) on an enamel sample, but did not affect the heights (*Sa*), voids (*Vvv*) or peak curvature (*Spc*). No significant changes in parameters were observed after treatment of enamel or dentine surfaces with phosphate buffer or water.

It should also be noted that we observed visual discolorations (Extended data fig. 2) of the samples irrespective of the treatment that was performed, including for samples

submerged in water, presumably due to the removal of small sediment particles adhering to the material. It therefore remains unclear if and to which extent color changes may also be induced by the reagents used.

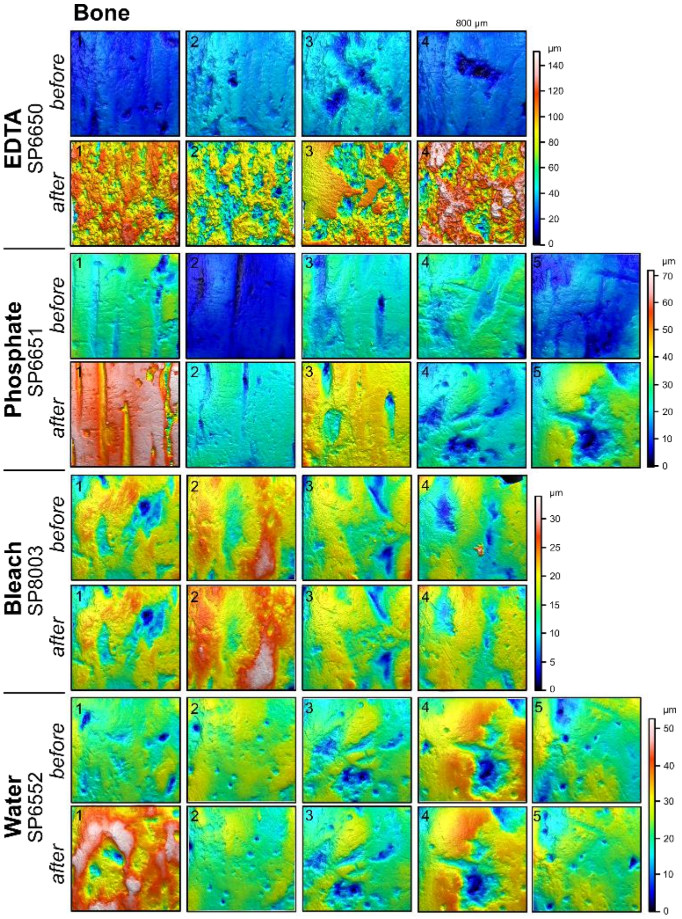

Supplementary figure 2.1. 3DST images of bones before and after treatment. Three-dimensional images of 3D surface texture measurements of bone samples. Note that the z-scale is consistent within each sample (before and after treatment), but not across samples due to the different surface topographies.

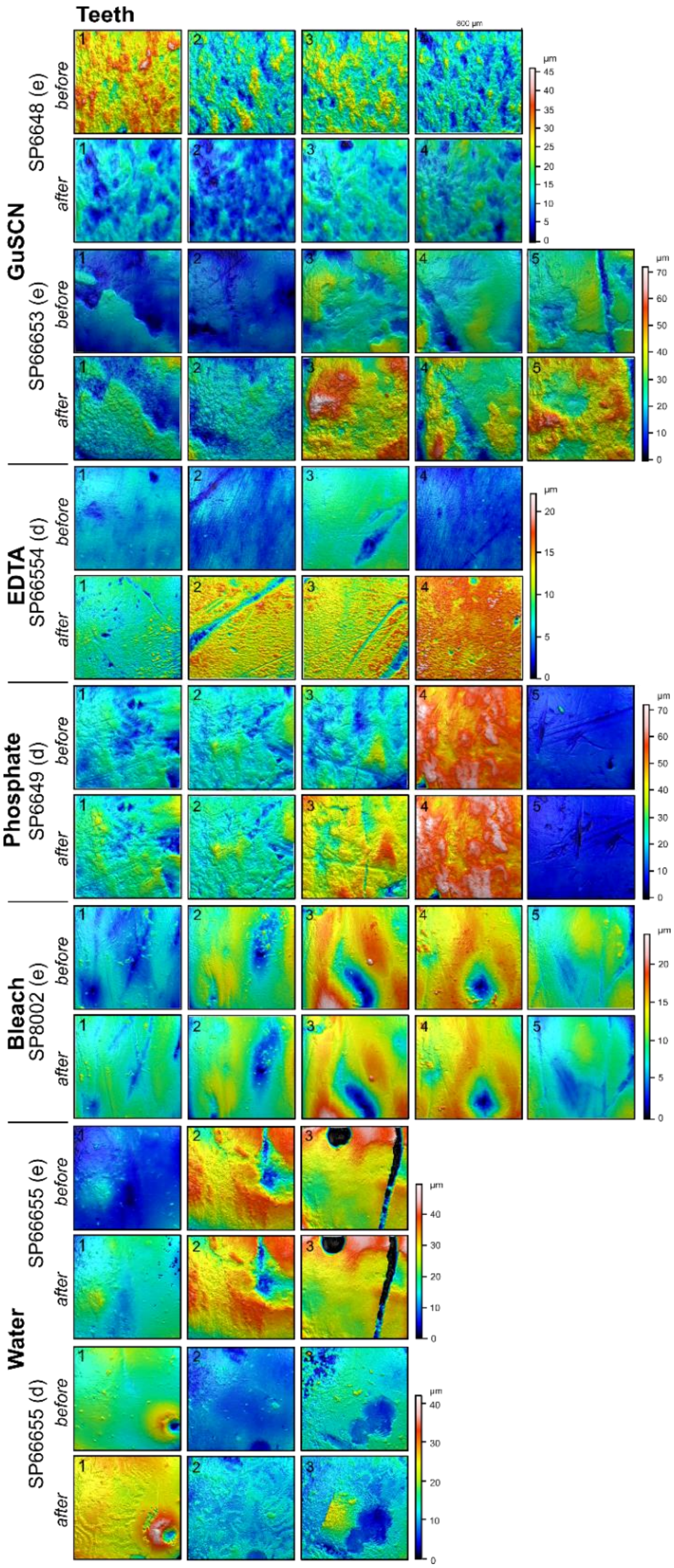

Supplementary figure 2.2. 3DST images of teeth before and after treatment. Three-dimensional images of 3D surface texture measurements of teeth samples. Note that the

z-scale is consistent within each sample (before and after treatment), but not across samples due to different surface topographies.

Supplementary table 2.1. Summary statistics of 3DST measurements. A two-sided paired t-test, before and after the treatment ($\alpha \leq 0.01$) was performed, mean and standard deviation (SD) of the measured surface texture parameters: *Sa* (arithmetical mean height), *Spc* (arithmetic mean peak curvature), *Spd* (density of peaks), *Vvv* (void volume of the valleys). Measurements were abbreviated as follows: R = before and T = after applying the reagent on tooth (d = dentine and e = enamel) and bone; N = number of measurements per spot. DNA extraction reagents were abbreviated by GuSCN = guanidine thiocyanate reagent, EDTA = ethylenediaminetetraacetic acid, phosphate = sodium phosphate buffer, bleach = sodium hypochlorite solution, water = distilled water.

**Specimen**	**Buffer**	**Material**	**N**	***Sa*** [µm]			***Spc*** [1/µm]			***Spd*** **[**1/µm^2**]**			***Vvv*** [µm^3/µm^2]		
				Mean	*SD*	*p*	Mean	*SD*	*p*	Mean	*SD*	*p*	Mean	*SD*	*p*
SP6648_R-d	GuSCN	tooth (d)		3.42	0.21	**0.009**	0.17	0.01	0.363	0.00024	0.00006	0.015	0.48	0.04	0.027
SP6648_T-d	GuSCN	tooth (d)	4	2.20	0.24		0.11	0.11		0.00006	0.00002		0.34	0.05	
SP6649_T-d	phosphate	tooth (d)		3.41	1.37	0.126	0.13	0.03	0.809	0.00004	0.00002	0.363	0.58	0.19	0.243
SP6649_R-d	phosphate	tooth (d)	5	3.89	1.85		0.14	0.05		0.00007	0.00006		0.65	0.26	
SP6654_R-d	EDTA	tooth (d)		0.64	0.18	0.010	0.08	0.02	**0.0001**	0.00050	0.00042	0.383	0.13	0.06	0.026
SP6654_T-d	EDTA	tooth (d)	4	1.35	0.40		0.18	0.02		0.00063	0.00019		0.29	0.12	
SP6655_R-e	water	tooth (e)		4.97	2.83	0.323	0.18	0.04	0.323	0.00007	0.00007	0.205	1.45	1.38	0.317
SP6655_T-e	water	tooth (e)	3	5.32	3.11		0.27	0.12		0.00003	0.00003		1.67	1.64	
SP6655_R-d	water	tooth (d)		1.76	0.66	0.096	0.21	0.08	0.389	0.00013	0.00006	0.238	0.32	0.17	0.678
SP6655_T-d	water	tooth (d)	3	2.12	0.84		0.28	0.09		0.00024	0.00018		0.35	0.12	
SP6653_R-e	GuSCN	tooth (e)		4.23	1.03	**0.002**	0.09	0.03	**0.005**	0.00002	0.00001	0.020	0.63	0.17	0.025
SP6653_T-e	GuSCN	tooth (e)	5	6.29	1.69		0.21	0.04		0.00007	0.00004		0.90	0.26	
SP8002_R-e	bleach	tooth (e)		1.99	0.80	0.290	0.12	0.02	0.717	0.00010	0.00005	0.010	0.37	0.18	0.303
SP8002_T-e	bleach	tooth (e)	5	1.90	0.69		0.12	0.03		0.00006	0.00004		0.36	0.17	
SP6650_R	EDTA	bone		3.46	1.00	**0.002**	0.11	0.03	**0.002**	0.00004	0.00001	0.041	0.90	0.40	**0.003**
SP6650_T	EDTA	bone	4	16.89	2.89		0.68	0.10		0.00010	0.00002		2.81	0.46	
SP6651_R	phosphate	bone		2.77	0.78	0.018	0.12	0.06	0.523	0.00004	0.00002	0.175	0.49	0.18	**0.008**
SP6651_T	phosphate	bone	5	4.02	1.32		0.11	0.03		0.00002	0.00001		1.03	0.42	
SP6652_R	water	bone		3.67	1.49	0.459	0.06	0.01	0.068	0.00002	0.00001	0.719	0.76	0.34	0.755
SP6652_T	water	bone	5	4.22	1.83		0.10	0.04		0.00001	0.00000		0.79	0.27	
SP8003_R	bleach	bone		3.19	0.16	0.637	0.15	0.05	0.074	0.00002	0.00001	0.184	0.59	0.06	0.417
SP8003_T	bleach	bone	4	3.11	0.34		0.09	0.02		0.00003	0.00001		0.54	0.07	

SI3: Compatibility of the method with radiocarbon dating

To ensure that Tween-20, a carbon-containing detergent ($C_{58}H_{114}O_{26}$) added at low concentration (0.1%) to the buffer used for non-destructive DNA extraction, would not compromise subsequent ^{14}C dating attempts, we carried out a series of tests. We selected a range of archaeological skeletal elements (bone/tooth dentine) for DNA and collagen extraction (Supplementary table 3.1). R-EVA 1658, 800, 123, 124 and 2907 were chosen as they had been previously pretreated and radiocarbon dated in methodological tests at the MPI-EVA (reported in[41,42]). From each skeletal element, two samples (A and B) were removed (ranging from 110 - 540 mg) using a diamond cutting disc. Non-destructive DNA extraction was carried out on sample A using the method described in the methods section, and on sample B using the same method but omitting Tween-20 from the phosphate buffer. For samples R-EVA 3723, 3724 and 3726, an additional sample C was taken and directly pretreated for ^{14}C dating without DNA extraction, to serve as a control.

Following the DNA extraction, collagen extraction was carried out in the Department for Human Evolution at the MPI-EVA using the bone pretreatment protocol for ^{14}C dating described in Fewlass et al. (2019)[42]. Four collagen extracts were selected for ^{14}C dating. These were sent to the Lab of Ion Beam Physics at ETH-Zurich (Switzerland) for graphitisation using the AGE3 equipment[43] and dating using the MICADAS AMS[44].

Routine quality checks were carried out on all collagen extracts: to obtain stable isotopic and elemental values, ~0.5 mg collagen was weighed into a tin cup and analyzed on a ThermoFinnigan Flash elemental analyzer (EA) coupled to a Thermo Delta plus XP isotope ratio mass spectrometer (IRMS). Stable carbon isotope ratios were expressed relative to VPDB (Vienna PeeDee Belemnite), and stable nitrogen isotope ratios were measured relative to AIR (atmospheric N_2), using the delta notation (δ) in parts per thousand (‰). Analysis of internal (Methionine EVA-12: $\delta^{13}C = -28.01 \pm 0.1$‰ (1SD) and $\delta^{15}N = -6.54 \pm 0.12$‰ (1SD)) and international standards (IAEA N1: $\delta^{15}N = 0.43 \pm 0.04$ ‰ (1SD), N2: $\delta^{15}N = 20.41 \pm 0.18$ ‰ (1SD), CH6: $\delta^{13}C = -10.45 \pm 0.3$ ‰ (1SD), CH7: $\delta^{13}C = -32.15 \pm 0.11$ ‰ (1SD)) indicates an analytical error of ± 0.3‰ (1σ) or lower. Extracts were further analyzed with Fourier Transform Infrared (FTIR) spectroscopy which is used to

characterize the quality of extracted collagen[45-47]. Roughly 0.3 mg of each extract was homogenized and mixed with ~40 mg of IR grade potassium bromide (KBr) powder in an agate mortar and pestle, pressed into a pellet using a manual hydraulic press (Wasserman) and analyzed with an Agilent Technologies Cary FTIR Spectrometer with a DTGS detector. Spectra were recorded in transmission mode at 4 cm^{-1} resolution with averaging of 34 scans between 4000 and 400 cm^{-1} using Resolution Pro software (Agilent Technologies, version 5.3.0.1964). The sample spectra were analyzed and compared to library spectra of well-preserved collagen and bone.

The collagen was well preserved in all samples (Supplementary table 3.1), with yields well above the minimum limit of 1% collagen. The yield values were consistent between extracts which had undergone phosphate-based non-destructive DNA extraction and those which had not, indicating that the DNA extraction did not significantly affect collagen preservation. The elemental values are also in agreement between all of the extracts from the same bone, falling within the ranges accepted for well-preserved collagen (C%: 30-50%, N%: 11-17% C:N: 2.9-3.6), which are commonly used to assess evidence of contamination and degradation[48]. The sample FTIR spectra are also characteristic of archaeological collagen extracts with no indication of large-scale contamination in any of the extracts. Radiocarbon dating of the four extracts which had undergone DNA extraction is consistent with previous dates obtained on the same bones, with three dating beyond the limit of the ^{14}C method (R-EVA 2907 is an MPI-EVA ^{14}C lab background bone dating to >50,000 BP), clearly demonstrating that no modern carbon contamination was present in the extracts, and a mammoth bone (35 ka) which has been dated extensively with different pretreatment methods[41,42,49,50]. Overall, there is no evidence of significant carbon contamination introduced by the Tween-20 used in the phosphate-based non-destructive DNA extraction method.

Supplementary table 3.1. Collagen yields and radiocarbon dates obtained from samples undergoing non-destructive DNA extraction with and without Tween-20. Pretreatment and ^{14}C dates from bone and dentine samples undertaken to determine if ^{14}C dating can be reliably carried out following phosphate-based non-destructive DNA extraction (Method A = standard method, B = no Tween-20, C = untreated control). Collagen extraction was performed for all samples, ^{14}C dates were produced for a sub-set of samples.

R-EVA	Sample ID	Method	Element	Sampled [mg]	Collagen yld [mg]	Collagen yld [%]	δ13 C [‰]	δ15 N [‰]	%C	%N	C: N	FTIR comment	AMS lab no.	F14C	error	14C age (BP)	error
**3723.1**	SP3385	A	Cave bear tooth root	403.1	47.6	11.8	-19.0	6.1	43.5	15.7	3.2	Collagen	ETH-118374	0.0001	0.0004	>50000	
**3723.2**	SP3385	B	Cave bear tooth root	541.2	60.9	11.3	-18.9	6.1	44.2	16.0	3.2	Collagen	ETH-118375	0.0002	0.0004	>50000	
**3723.3**	SP3385	C	Cave bear tooth root	423	51.5	12.2	-19.0	5.8	40.8	15.4	3.1	Collagen					
**3724.1**	SP3386	A	Cave bear tooth root	441.8	54.1	12.2	-19.3	7.8	46.4	16.1	3.4	Collagen					
**3724.2**	SP3386	B	Cave bear tooth root	166	20	12.0	-19.4	7.6	44.2	15.7	3.3	Collagen					
**3724.3**	SP3386	C	Cave bear tooth root	259.9	33.3	12.8	-19.3	7.5	44.4	15.7	3.3	Collagen					
**3726.1**	SP3390	A	Cave bear tooth root	268.8	42.8	15.9	-19.5	7.9	44.6	16.1	3.2	Collagen					
**3726.2**	SP3390	B	Cave bear tooth root	212.5	27.8	13.1	-19.5	8.0	44.0	15.9	3.2	Collagen					
**3726.3**	SP3390	C	Cave bear tooth root	146.1	25.4	17.4	-19.6	8.2	44.2	16.0	3.2	Collagen					
**1658.1**	SP3391	A	Bovid bone	109.8	7.5	6.8	-19.4	5.5	41.2	14.8	3.2	Collagen					
**1658.2**	SP3391	B	Bovid bone	111.4	10.5	9.4	-19.5	5.4	42.2	15.3	3.2	Collagen					
**800.1**	SP3571	A	Cave bear long bone	328.9	20.2	6.1	-21.8	0.0	44.7	15.2	3.4	Collagen					
**800.2**	SP3571	B	Cave bear long bone	339.6	35	10.3	-21.7	0.0	47.2	16.0	3.4	Collagen					
**123.1**	SP4052	A	Mammoth rib	242.5	27.9	11.5	-21.6	7.1	43.6	15.6	3.3	Collagen	ETH-118376	0.0119	0.0004	35,610	270
**123.2**	SP4052	B	Mammoth rib	265.4	31.8	12.0	-21.5	7.1	44.0	15.8	3.3	Collagen					
**124.1**	SP4053	A	Woolly rhino long bone	476	37.2	7.8	-20.6	3.3	44.2	15.8	3.3	Collagen					
**124.2**	SP4053	B	Woolly rhino long bone	396.5	36.9	9.3	-20.6	3.5	44.3	15.9	3.3	Collagen					
**2907.1**	SP11371	A	Cave bear long bone	153.3	8.1	5.3	-20.5	5.3	43.1	15.5	3.3	Collagen	ETH-118377	0.0001	0.0004	>50000	
**2907.2**	SP11371	B	Cave bear long bone	200.7	10.6	5.3	-20.4	5.4	41.9	14.8	3.3	Collagen					

SI4: Taxonomic assignment of mtDNA sequences

Taxonomic assignment of sequences resulting from mtDNA capture and the evaluation of the presence of ancient DNA damage patterns were performed as described in the Methods section (using a previously published computational pipeline[51] based on BLAST and MEGAN[52], with modifications in data filtering detailed in Vernot et al. (2021)[53]). Summary statistics of sequencing (generated using 'samtools'[54] version 1.3.1) and the identification of ancient mammalian taxa are provided in Data file S1. The numbers of mtDNA fragments released from the six artefacts that yielded ancient DNA are plotted in Fig. 2, together with their taxonomic assignments. The dominant ancient mammalian families identified in the phosphate fractions of these artefacts match the morphology-based taxonomic identification of the artefacts where available (Extended data table 1), with exception of the first room temperature phosphate fraction from BKP2, which yielded slightly more bovid (n = 619) than ursid (n = 458) mtDNA fragments (Data file S1). Furthermore, for all freshly excavated artefacts except BKP1, the dominant ancient mammalian families recovered from the attached sediment differ from those in the phosphate fractions, indicating that phosphate-based non-destructive DNA extraction is suitable, in most cases, for determining the species the artefacts originated from. Supplementary fig. 4.1 shows the yield of mtDNA from the nine artefacts from which no ancient DNA was recovered.

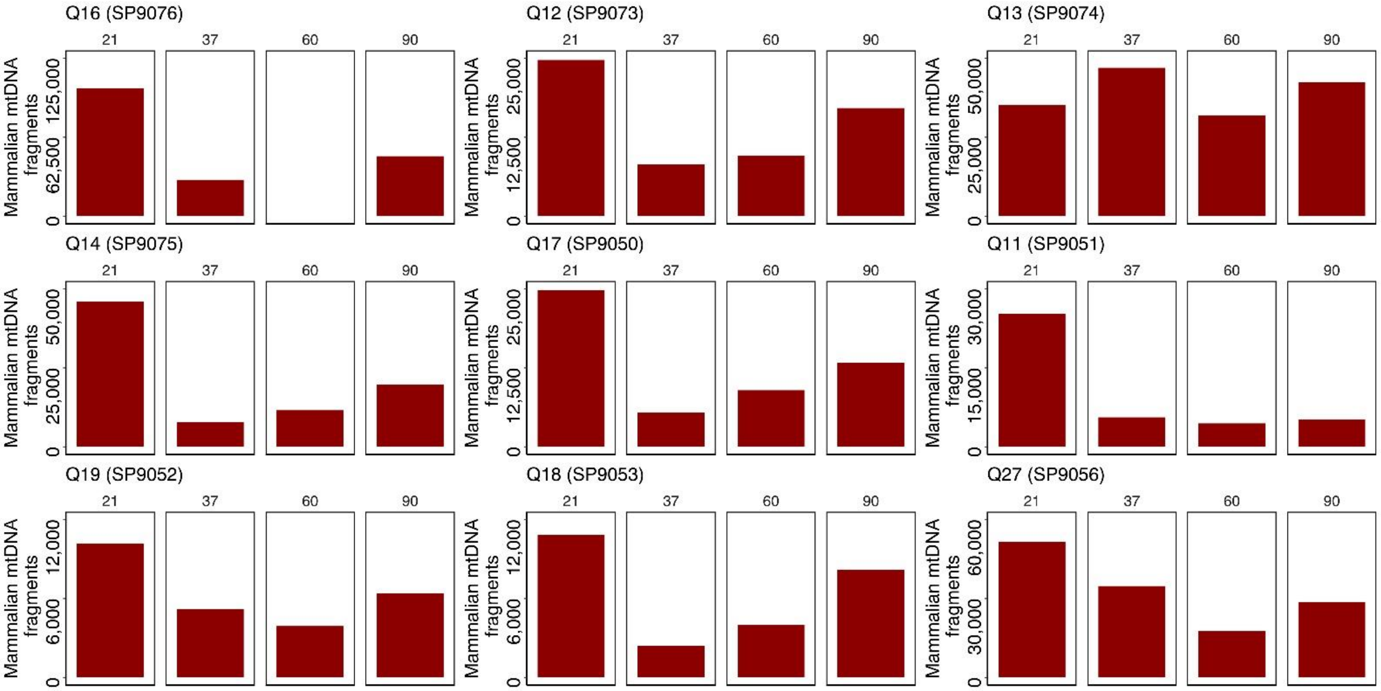

Supplementary figure 4.1. Release of mammalian mtDNA. Mammalian mitochondrial DNA released from samples that did not show evidence for the presence of ancient mammalian DNA. Continuation of Fig. 2. in main text. Identified taxa include *Hominidae, Suidae* and *Bovidae* (see Data file S1).

SI5: Denisova Cave pendant – human mtDNA analysis

Patterns of cytosine deamination and estimates of present-day human contamination

Seven fractions of DNA recovered from the Denisova pendant (the first and second sediment pellet, the first 37 °C phosphate fraction, the first 60 °C phosphate fraction and all three 90 °C phosphate fractions) yielded ancient human mtDNA. While the numbers of mtDNA fragments recovered from the sediment pellets and the first fractions of the 37 °C, 60 °C and 90 °C phosphate incubations ranged between 128 and 1,574 fragments only (or between 0.4 and 6.0-fold coverage of the mitochondrial genome), 4,315 and 5,186 fragments (amounting to 13.2 and 15.9-fold coverage) were recovered from the second and third 90 °C phosphate fractions, respectively (Data file S1). To further increase the sequence coverage of the mtDNA genome, we produced four additional libraries from each the second and third 90 °C phosphate fractions, increasing the total coverage to 61.6 and 84.1-fold, respectively (Data file S1 and Supplementary table 5.1).

To estimate the contribution of present-day human DNA contamination to these data we first compared the frequencies of deamination-induced terminal 5' and 3' C-to-T substitutions in all DNA fragments from each fraction to those in fragments carrying a C-to-T substitution on the opposing end ('conditional substitution frequencies'[55]). The point estimates of the C-to-T substitution frequencies fall within the 95% binomial confidence intervals of the conditional substitution frequencies in all six fractions (Supplementary table 5.1), providing no evidence for the presence of a mixture of molecules with different deamination rates as would be expected if the libraries were contaminated with present-day human DNA. However, the large confidence intervals of the conditional substitution frequencies indicate that this analysis does not provide enough power for detecting low or moderate levels of contamination. We therefore used AuthentiCT, a software tool that uses a more complex model of post-mortem DNA damage, for estimating the proportion of present-day human contamination in the data[56]. Based on this model, the second 90 °C phosphate fraction is estimated to contain only 0.1% contamination (95% confidence interval: 0 - 2.8%), whereas contamination is estimated to >5% in all other fractions (Supplementary table 5.1).

Number of mtDNA haplotypes and consensus calling

We next aimed to determine whether the human mtDNA fragments recovered from the pendant represent a single haplotype, as would be expected if the DNA belonged to a single individual. Evaluating the consistency of bases at each position in the mitochondrial genome requires high coverage and low present-day human contamination, criteria that are best met by the second 90 °C phosphate fraction. We therefore determined the majority base at each position in the mitochondrial genome covered by at least 10 mtDNA fragments obtained in this fraction. The coverage filter excluded 237 bp (1.4%) of the genome, which are located at the beginning and the end of the reference genome and in four small contiguous regions in the 12S rRNA, 16S rRNA and tRNA-Met genes (Supplementary fig. 5.1). The lack of coverage in these regions is presumably due to high evolutionary sequence conservation that makes it difficult to distinguish human sequences from those of other mammals.

After manually correcting alignment problems around a C-homopolymer stretch (positions 303-315 of the reference genome) and a dinucleotide deletion present in all sequenced fragments (positions 514-515), we determined the support for the majority (consensus) base at each of the 16,332 positions covered by 10 or more fragments. To mitigate the impact of deamination, T's occurring in the first three or last three positions of each fragment in the orientation as read were disregarded in this analysis. Only three positions showed a consensus support of less than 80%, and none less than 74% (Supplementary fig. 5.1), suggesting that we obtained a consensus sequence from predominantly one haplotype, but that one or more other haplotypes may additionally be present at low frequency. Haplogroup assignment of the consensus sequence using Haplogrep 2 (version2.4.0)[57] showed the highest support for haplogroup U (overall quality 0.85), with all three mutations present that differentiate haplogroup U from the more basal R haplogroup.

It is possible that the composition of mtDNA haplotypes differs among the fractions of DNA released from the Denisova Cave pendant. To determine if the dominant haplotype from the second 90 °C fraction is also dominant in the other fractions, we aligned the consensus sequence to the mtDNA sequences of a world-wide panel of 311 human

individuals[58]. This allowed us to identify seven 'diagnostic' positions in the mitochondrial genome at which the consensus sequence of the second 90 °C fraction differs from at least 99% of the haplotypes of other humans (as well as one insertion/deletion, which was not used in subsequent analysis; Extended data table 2). We then identified all mtDNA fragments overlapping these positions in each DNA fraction recovered from the pendant and determined the proportion of fragments that matched the consensus base of the second 90 °C phosphate fraction (masking T's in the first and last three positions of each fragment to minimize the impact of deamination). The support of the consensus base ranged between 77.8 and 86.6% in the three 90 °C phosphate fractions, albeit with large confidence intervals in the first fraction (Extended data table 3). These results suggest that 78% or more of the sequences released in the highest temperature incubations belong to the dominant haplotype from which the consensus sequence was reconstructed. The fact that the consensus support does not exceed 86.6% (95% binomial confidence interval (C.I.): 82.2 - 90.5) in the second 90 °C phosphate fraction, which shows close to zero evidence for present-day human contamination, indicates that the haplotype(s) present in the data at minor frequencies originate, at least partly, from one or more additional ancient individuals.

The mtDNA fragments recovered from the first and the second sediment pellet as well as the 60 °C phosphate fraction showed only low support for the dominant haplotype present in the 90 °C phosphate fractions (20.0% (95% C.I.: 5.7 – 43.7), 9.5% (95% C.I.: 1.2 – 30.4) and 37.5% (95% C.I.: 8.5 – 75.5), respectively; Extended data table 3). The conditional substitution patterns and AuthentiCT suggest that all three fractions are dominated by ancient DNA, indicating that the low support for the haplotype from the second 90 °C phosphate fraction cannot be explained solely by contamination with present-day human DNA. Restricting the analysis to only deaminated fragments retained only six fragments overlapping a diagnostic position: four in the first sediment pellet, one in the second, and one in the 60 °C fraction. Four of these fragments support the state shared by present-day humans. No diagnostic positions were covered in the 37 °C fraction. Taken together these results suggest that the ancient human mtDNA that was released in the 90 °C phosphate fractions is dominated by one haplotype, and that the

preceding fractions, including the sediment pellets, are dominated by one or more different haplotypes from other ancient human individuals.

Tree building and molecular branch shortening

The pendant was discovered in layer 11 of the South Chamber, a portion of the stratigraphy that spans a time period of at least ~24-39 ka (Supplementary information 1). Therefore, in order to place the reconstructed mtDNA genome within the context of currently published present-day and ancient human mtDNA genomes and to better understand the temporal context, we attempted tree building and genetic dating using BEAST2 (version 2.6.6)[59]. Using MAFFT (version 7.453)[60] we aligned the newly reconstructed mtDNA genome from the pendant to the mtDNA sequences of 54 modern humans from populations around the world[61], 19 ancient modern humans (Supplementary table 5.2), the rCRS[62] and the Vindija 33.16 Neandertal[58], which was used as an outgroup. After alignment all ambiguous positions were removed.

For the molecular branch shortening and tree building, BEAST2 (version 2.6.6), a Bayesian framework, was used as described below. To determine the best fitting clock and tree models, a path sampling approach from BEAST2's MODEL_SELECTION package[63-65] was used with the Tamura-Nei 1993 (TN93)[66] substitution model for all model combinations. We tested both strict and relaxed log normal clock models with constant and Bayesian skyline tree models. For the path sampling, 40 path steps with 25,000,000 iterations were used with an alpha parameter of 0.3, pre-burn-in of 75,000 iterations and an 80% burn-in of the complete chain. For each clock model a mutation rate of 2.53×10^{-8} was used with a normal distribution and a sigma of 1.00×10^{-10} [67]. The tree was calibrated using previously published dates for ancient modern humans and Vindija 33.16, and using the updated IntCal20[12] (see Supplementary table 5.2 for dates and individuals used). Ancient modern humans of unknown date (including the newly reconstructed mtDNA genome) were constrained to a range from 0 to 60,000 years and all present-day humans were set to present day (date = 0).

While the model with a relaxed log normal clock and tree with a constant population size was a worse fit to the data than both models with a Bayesian skyline population tree model (log10 Bayes Factor > 3.2), there was no significant difference among the other

clock and tree model combinations (Supplementary table 5.3). Thus, we tested the remaining three models (bold in Supplementary table 5.3). We ran three Markov chain Monte Carlos (MCMC) with 75,000,000 iterations, sampling every 2,000 trees and using a burn-in of 10,000,000 iterations for each model. BEAST2's logcombiner2 was used to combine the resulting log and tree files for each model, and BEAST2's treeannotator was used to annotate the merged tree file in a single tree. Figtree (v1.4.4, https://github.com/rambaut/figtree/) was used for tree visualization and BEAST2's Tracer program was used to examine the resulting tip dates (Supplementary figure 5.2). The model with a relaxed log normal clock and Bayesian skyline population had the lowest log likelihood (Supplementary table 5.3). For this model the mtDNA from the pendant was estimated to have a tip date of 18,513 ka (95% highest posterior density interval (HPDI): 4,579 to 31,562 years before present) (see Supplementary table 5.3 for estimates obtained with the other models).

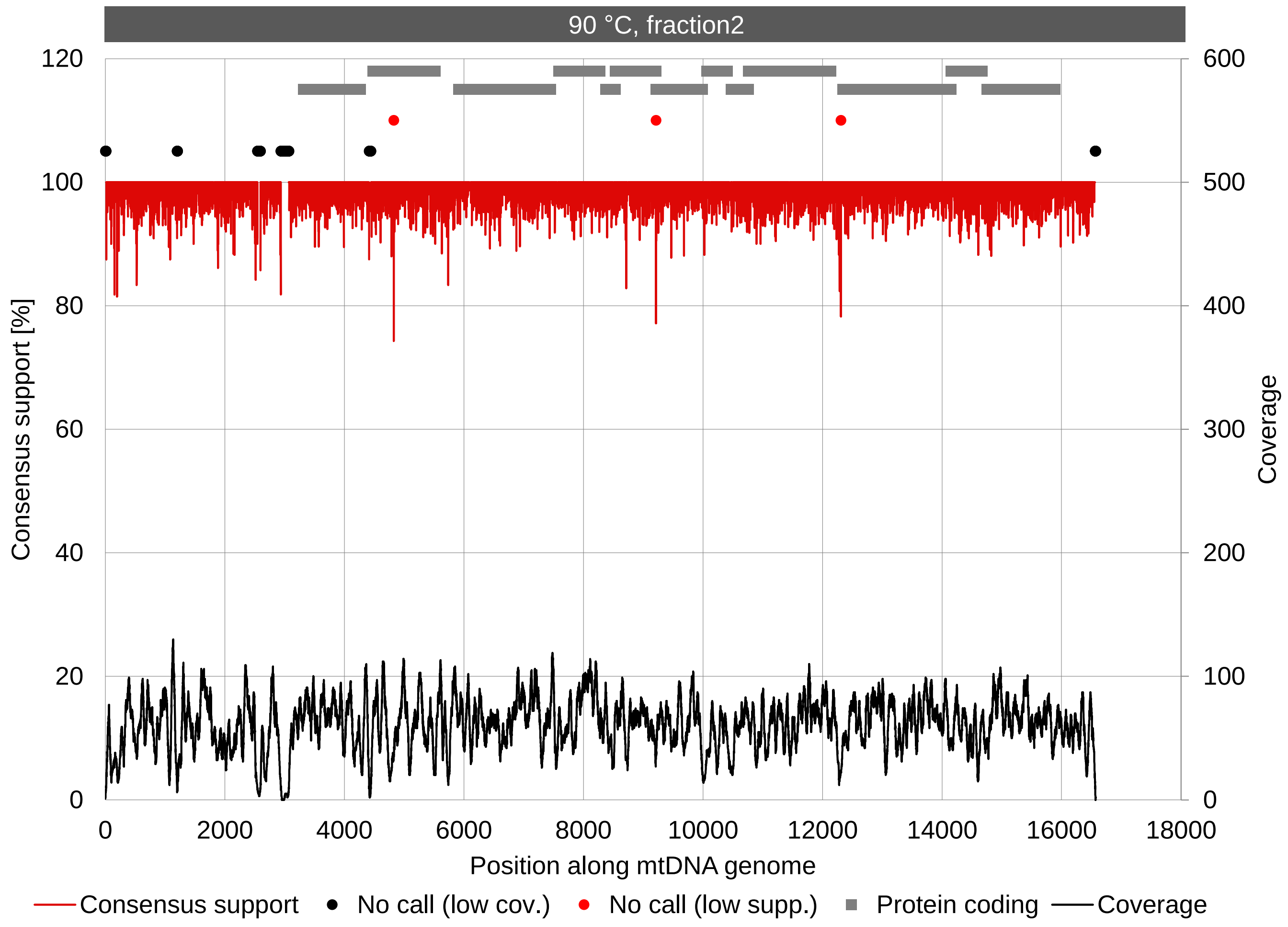

Supplementary figure 5.1. Human mitochondrial coverage and consensus support. Human mitochondrial coverage and consensus support obtained for the second 90 °C phosphate fraction recovered from the Denisova Cave pendant.

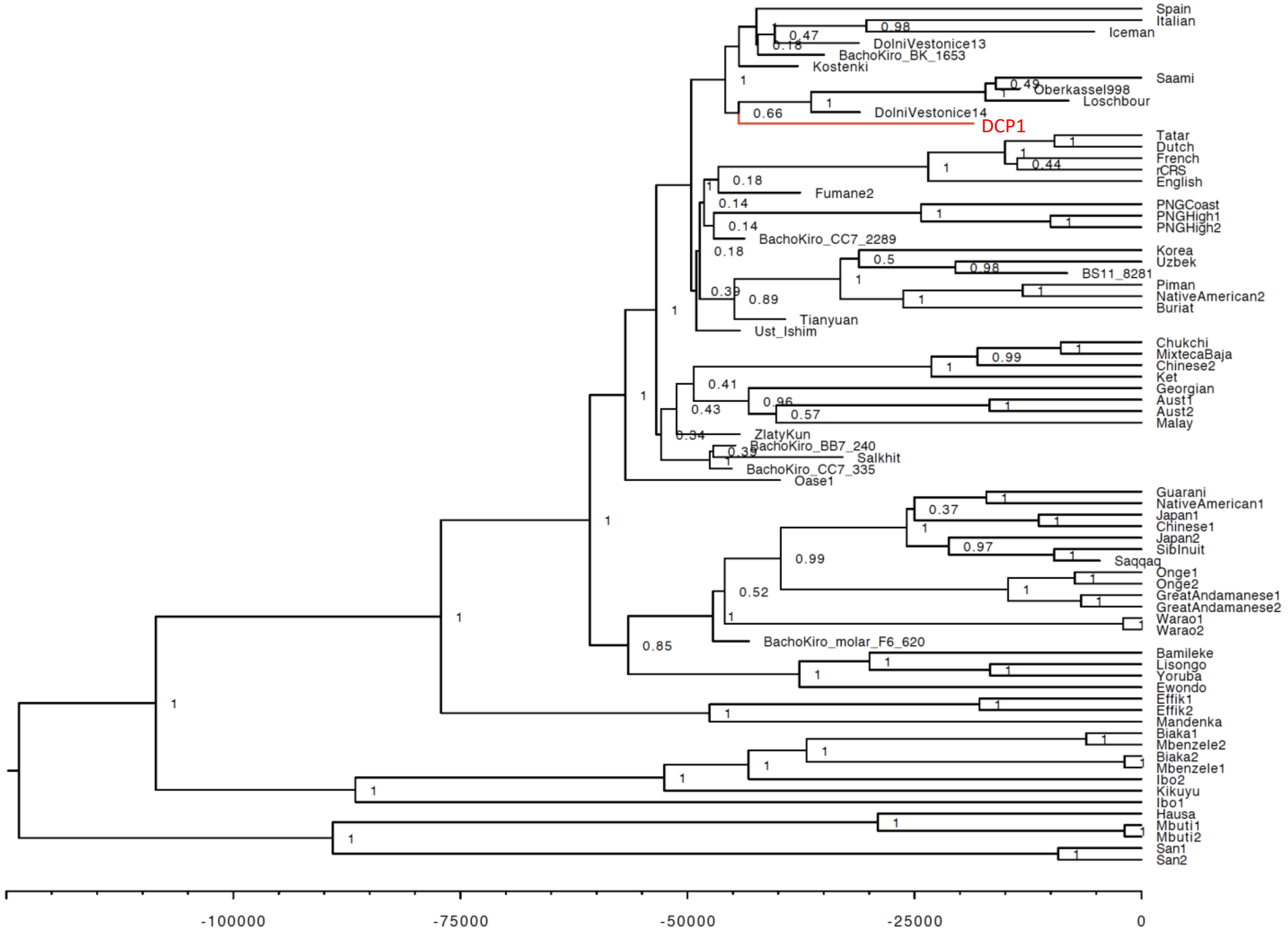

Supplementary figure 5.2. Bayesian human mtDNA tree including DCP1. Bayesian tree built with BEAST2 (version 2.6.6) depicting the relationship of the mtDNA sequence from the Denisova Cave pendant to other ancient and present day human mtDNA sequences. This tree was constructed using a relaxed log normal clock and Bayesian skyline population model. The nodes are labeled with the corresponding posterior probabilities. The x-axis represents years from present. The Neandertal mtDNA genome used to root the tree (Vindija 33.16) is not depicted.

Supplementary table 5.1. Summary statistics of human mtDNA recovered from DCP1. MtDNA yields, deamination patterns, and present-day human contamination estimates obtained for all DNA fractions from the Denisova Cave pendant containing ancient human DNA.

Fraction	#Libraries	Unique mtDNA fragments	MtDNA coverage	C-to-T substitution frequency [%] (95% conf. int.)		Conditional C-to-T substitution frequency [%] (95% conf. int.)		AuthentiCT present-day human contamination estimate [%] (95% conf. int.)
				5' end	3' end	5' end	3' end	
Sediment pellet 1	1	1,574	6.0	21.9 (17.9-26.3)	14.2 (10.7-18.3)	43.8 (19.8-70.1)	26.9 (11.6-47.8)	40.0 (34.2-45.8)
Sediment pellet 2	1	827	2.6	35.0 (28.4-42.0)	18.3 (13.1-24.4)	11.1 (0.3-48.2)	7.7 (0.2-36.0)	5.6 (0.0-20.6)
37 °C, fraction 1	1	128	0.4	33.3 (16.5-54.0)	31.7 (18.1-48.1)	40.0 (5.3-85.3)	66.7 (9.4-99.2)	25.1 (0.0-50.3)
60 °C, fraction 1	1	472	1.5	16.7 (10.5-24.6)	20.0 (13.1-28.5)	20.0 (0.5-71.6)	16.7 (0.4-64.1)	12.1 (0-29.1)
90 °C, fraction 1	1	727	2.2	28.3 (21.9-35.5)	19.1 (13.7-25.6)	23.1 (5.0-53.8)	18.8 (4.0-45.6)	12.8 (1.0-24.6)
90 °C, fraction 2	5	20,875	61.6	29.5 (28.4-30.7)	19.6 (18.5-20.6)	29.3 (24.5-34.5)	18.6 (15.3-22.1)	0.1 (0.0-2.8)
90 °C, fraction 3	5	27,934	84.1	27.2 (26.3-28.2)	19.0 (18.1-19.9)	30.0 (25.8-34.4)	20.7 (17.7-23.9)	6.6 (4.3-8.9)

Supplementary table 5.2. List of individuals used for tree-building and molecular branch shortening. Previously published ancient modern human and Neandertal mtDNA genomes used for tree building and molecular branch shortening. Dates listed were calibrated using IntCal20[12] and OxCal4.4[13].

Individual	Radiocarbon Date (INTCAL20, 95.4% confidence interval)	Country	Reference for mtDNA
**Fumane2**	NA	Italy	Benazzi et al., 2015[68]
**Oase1**	37,950 - 41,860	Romania	Fu et al., 2015[69]
**Ust'Ishim**	42,900 - 45,930	Russia	Fu et al., 2014[70]
**Boshan 11**	8,030 - 8,320	China	Fu et al., 2013[67]
**Loschbour**	7,930 - 8,170	Luxembourg	Fu et al., 2013[67]
**Tianyuan**	38,070 - 40,850	China	Fu et al., 2013[71]
**Kostenki 14**	36,670 - 39,380	Russia	Krause et al., 2010[72]
**Iceman**	4,970 - 5,450	Italy	Ermini et al., 2008[73]
**Saqqaq**	4,410 - 4,790	Greenland	Gilbert et al., 2008[74]
**Oberkassel**	13,180 - 13,730	Germany	Fu et al., 2013[67]
**Dolni Vestonice 14**	30,860 - 31,150	Czech Republic	Fu et al., 2013[67]
**Dolni Vestonice 13**	31,030 - 31,240	Czech Republic	Fu et al., 2013[67]
**Salkhit**	32,390 - 33,440	Mongolia	Deviese et al., 2019[75]
**ZlatyKun**	NA	Czech Republic	Prufer et al., 2021[76]
**Bacho Kiro F6_6**	42,580 - 43,930	Bulgaria	Hublin et al., 2020[8]
**Bacho Kiro BB7_240**	43,940 - 45,550	Bulgaria	Hublin et al., 2020[8]
**Bacho Kiro CC7_335**	44,420 - 45,930	Bulgaria	Hublin et al., 2020[8]
**Bacho Kiro CC7_2289**	42,990 - 44,400	Bulgaria	Hublin et al., 2020[8]
**Bacho Kiro 1653**	34,610 - 35,290	Bulgaria	Hublin et al., 2020[8]
**Vindija 33.16**	44,690 - 49,930	Croatia	Green et al., 2008[74]

Supplementary table 5.3. Overview of log likelihoods for different clock models. The estimated log likelihoods for different tree and clock models using a path sampling approach.

Clock Model	Tree Model	Marginal log likelihood	Tip Date (years before present)	95% HPDI (years before present)
**Strict**	**Constant**	-30,744.78	16,769	2,969 - 29,891
**Strict**	**Bayesian skyline**	-30,738.06	17,826	4,358 – 30,245
Relaxed Log Normal	Constant	-30,745.53	NA	NA
**Relaxed Log Normal**	**Bayesian skyline**	-30,737.89	18,513	4,579 – 31,562

SI6: Denisova Cave pendant – cervid mtDNA analysis

Consensus calling

In an initial characterization of the DNA fractions recovered from the pendant, a mammalian capture probe set was used that is composed of the mtDNA genomes from 242 mammals[77] (see Methods section and Supplementary information 4). This probe set is well suited for determining the presence of ancient DNA from a wide range of mammals, but produces non-uniform sequence coverage across the mtDNA genome. As the pendant had been identified as a cervid tooth, we selected the library with the highest proportion of cervid DNA (97.4%; library A34688 from the first 60 °C fraction; see Data file S1) and enriched it with a cervid mtDNA probe set available in our laboratory. This probe set (‘AA101’) was originally designed for another study and contains 52-mer probes covering three cervid mtDNA genomes (*Cervus elaphus*, *Capreolus capreolus* and *Dama dama*; NCBI GenBank accession numbers AB245427, JN632610 and NC_020700) in 1 bp tiling. Eight-base-pair linker sequences were added to each probe (for a total length of 60 bp) and the probes were printed on 1M Sure Select DNA Capture arrays (Agilent Technologies), stripped and converted into probe libraries as described in detail elsewhere[71]. Hybridization capture, sequencing and data processing (including the identification of cervid mtDNA sequences) were then performed exactly as described in the Methods section and Supplementary information 4.

More than 99% of the mtDNA fragments that were identified on the biological family level were assigned to *Cervidae*. In addition, preliminary analyses indicated that the mtDNA fragments belonged to a wapiti (*Cervus canadensis*), an elk native to North America and Central and East Asia. We therefore dropped the metagenomics filtering and mapped all sequences directly to a complete mtDNA genome from this species (NCBI GenBank accession number NC_050863[78]) using BWA (version 0.5.10-evan.9-1-g44db244)[79] with ‘ancient parameters’ (‘-n 0.01 –o 2 –l 16500’)[80]. Unmapped sequences, sequences mapping with a map quality score of less than 25 and sequences shorter than 35 bp were discarded. After duplicate removal using bam-rmdup (https://github.com/mpieva/biohazard-tools/tree/v0.2-knowngood) , we obtained 168,631

unique mtDNA fragments, corresponding to 636-fold coverage of the genome (Supplementary table 6.1).

MtDNA consensus calling was performed as described in Supplementary information 5, with the difference of requiring at least 5-fold coverage and 80% consensus support at each position. A total of 191 positions failed the coverage threshold (Supplementary table 6.1 and Supplementary fig. 6.1), which were located in a region of the mtDNA genome where the wapiti reference genome shows multiple short insertion/deletions (indels) when aligned to the cervid genomes used for probe design and capture. The largest of these indels is an insert of 76 bp (positions 15603-15678 in the wapiti reference genome). Among the 16,371 positions that passed the coverage threshold (98.8% of the reference genome), only two showed a consensus support of less than 80% after manually correcting the consensus calls around a C-stretch where alignment problems had occurred (Supplementary fig. 6.1). One of these two unresolved positions showed a consensus support of 75% (6 C and 2 T), likely due to deamination. Here, we manually called C as consensus base. The other position (70% consensus support; 21 C and 9 T) remained unresolved and may reflect a heteroplasmy or the presence of mtDNA fragments from humans or another species falsely assigned to *Cervidae*.

Generating additional ancient wapiti mtDNA genomes

Although the mtDNA diversity of ancient and modern wapitis has been characterized in previous studies using the *cytb* gene and control region[81,82], no complete mtDNA genomes are available from ancient wapiti samples, limiting the utility of the complete mtDNA sequence we generated for genetic dating. We therefore obtained eight specimens for which radiocarbon dates (recalibrated here using IntCal20[12] and OxCal 4.4[13]) and *cytb* sequences were determined in a previous study[83] (Supplementary table 6.2) and prepared DNA extracts (from between 16 and 25 mg bone powder) as well as single-stranded libraries. We then enriched these libraries for the complete mtDNA genome using the cervid probe set and generated 76-bp paired end reads on a MiSeq sequencer (Illumina). The laboratory work and raw sequence data processing was performed using the methods detailed in the Methods section.

The number of unique mtDNA fragments obtained from the eight ancient cervid bones ranged from 49,041 to 376,190 (between 200 and 1,687-fold coverage of the genome) (Supplementary table 6.1). No cervid sequences were identified in the negative controls which had been carried through DNA extraction, library preparation, hybridization capture and sequencing. All samples displayed elevated frequencies of C-to-T substitutions at the molecule ends (Supplementary table 6.1), but less than 10% of terminal cytosines were converted to thymines in two of the youngest samples, dated to between 300 and 510 calibrated years before present (cal BP), respectively (Supplementary table 6.2).

Consensus sequences were called as described above. Positions with a consensus support of less than 80% (n = 30) were visually inspected and consensus bases called if the lack of support was due to alignment problems in the vicinity of homopolymer stretches (n = 16) or likely due to deamination close to the ends of molecules (n = 11). This left three ambiguous positions, two of which were covered by only five mtDNA fragments and one by 17 (Supplementary table 6.1). In addition, between 81 and 317 positions could not be called due to low coverage (most of them in proximity to the 76-bp insertion in the wapiti reference genome). In summary, high-quality mtDNA genomes were obtained from all eight samples, covering between 98.1 and 99.5% of the mtDNA genome.

<u>Tree building and genetic dating</u>

In order to place the mtDNA genome reconstructed from the pendant into context with the newly constructed complete wapiti mtDNA genomes we attempted tree building and molecular dating via Bayesian analysis with BEAST2 (version 2.6.6)[59]. We aligned these nine mtDNA genomes and a red deer (*Cervus elaphus,* NC_007704.2) mtDNA genome[84] to the wapiti mtDNA genome (GenBank accession number NC_050863) using MAFFT (version 7.453)[60]. The Tamura-Nei (TrN) model[66] was identified as the best nucleotide substitution model to use with this dataset by using jModelTest (version 2.1)[85]. To estimate a mutation rate for the full wapiti mtDNA genome we used the youngest seven of the newly generated genomes described above and followed the Bayesian approach used to estimate human mtDNA mutation rates[67]. The oldest sample resulted in a calibrated upper limit that was beyond the radiocarbon dating range (Supplementary table

6.2). In short, we used a path sampling approach from BEAST2 (version 2.6.6) to determine which clock (strict or relaxed) and tree (constant or exponential population growth) model were the best fit for the data. From this evaluation we determined that a strict clock with a constant population was the best fit (Supplementary table 6.3). With a strict clock and constant population, we performed two MCMC runs, each with 30,000,000 iterations, a burn-in of 6,000,000 and logging every 1,000 steps. The results of each run were then combined with the Logcombiner2 package from BEAST2 (version 2.6.6). The 95% highest posterior density of the estimated clock rate was 8.31 X 10^{-12} to 3.07 X 10^{-8} with a median of 9.6 X 10^{-9}. Using this median clock with a normal distribution prior, we repeated the path sampling model evaluation with the full set of ten mtDNA genomes to identify which combination of clock (strict or relaxed log normal) and tree (constant population or Bayesian skyline) was the best fit. A prior of 0 to 60,000 years ago was used for the date of the pendant mtDNA genome. The Bayesian skyline was identified as a better fit between the two tree types. There was no significant difference between the strict and relaxed clock with the Bayesian skyline tree model. Thus, we selected the strict clock as it is the simplest model. We then ran three MCMC runs with a strict clock model and the Bayesian skyline tree model and combined the resulting log and tree files with Logcombiner2. The combined tree was then annotated with Treeannotator from the BEAST2 package. The 95% highest posterior density interval for the Denisova pendant was 12,781 to 38,995 years ago with a mean age of 24,652 years before present. In the tree the cervid mtDNA genome of the pendant is closest to a wapiti mtDNA genome from Birukova, Russia (Supplementary fig. 6.2).

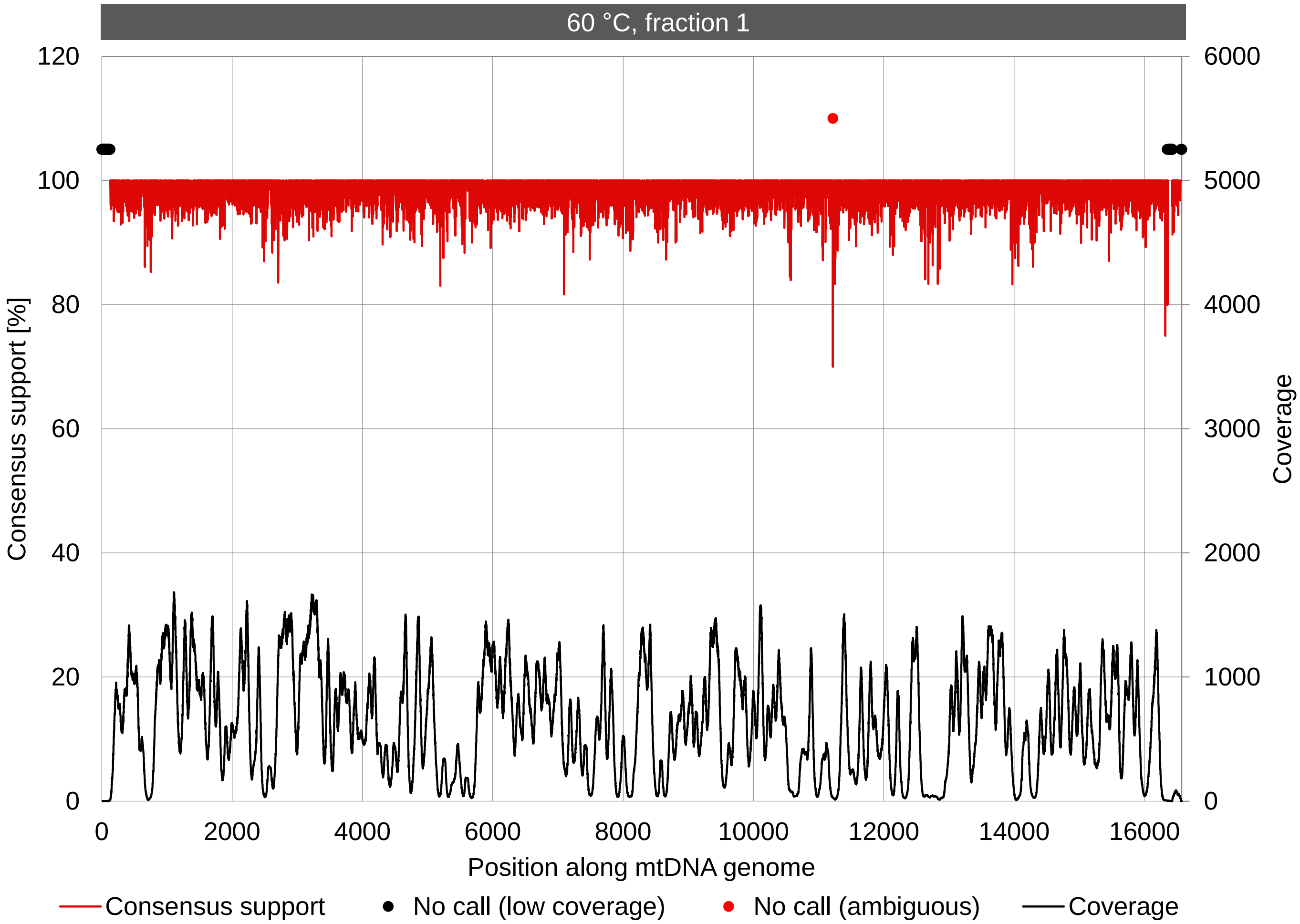

Supplementary figure 6.1. Wapiti mitochondrial coverage and consensus support. Mitochondrial coverage and consensus support obtained for the wapiti mtDNA genome reconstructed from the first 60 °C phosphate fraction of the Denisova Cave pendant.

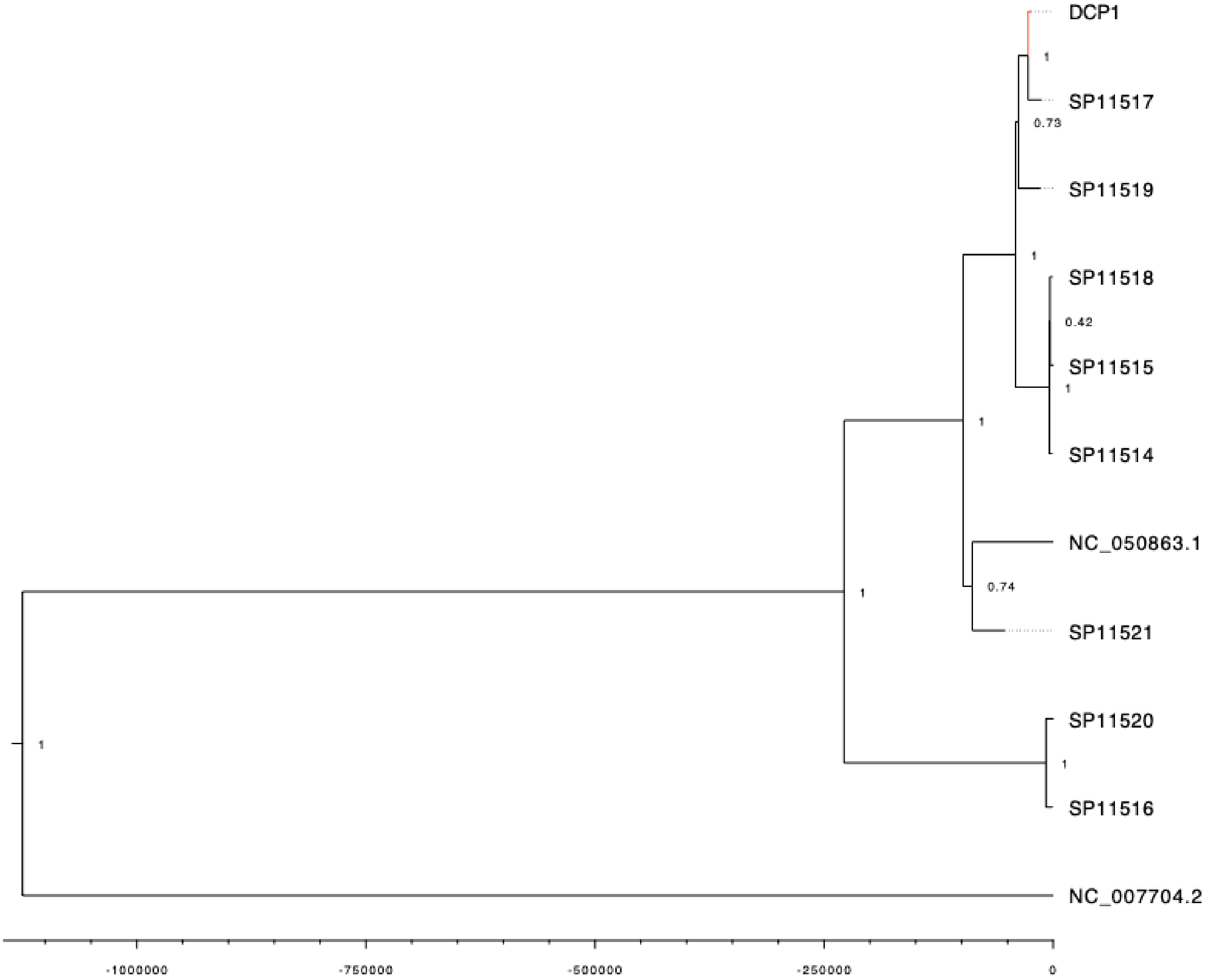

Supplementary figure 6.2. Bayesian tree including DCP1 wapiti mtDNA sequence. Bayesian phylogenetic tree of wapiti mtDNA genomes constructed using BEAST2 (version 2.6.6). The tree was rooted using a red deer sequence (NC_007704.2). The nodes are labeled with the corresponding posterior probabilities. The x-axis represents years from present.

Supplementary table 6.1. Summary statistics of wapiti mtDNA. Summary statistics describing the wapiti mtDNA fragments and consensus sequences recovered from the Denisova pendant and eight ancient wapiti bones.

MPI ID	Library ID (Cap. Library ID)	Sequences generated	Unique mapped sequences	C-to-T substitution frequency [%] (95% conf. int.)		----------------------MtDNA consensus calling ------------------			
				5' end	3' end	Coverage [-fold] (%genome)	Called	Low coverage (<5-fold)	Ambiguous (observed bases)
Denisova pendant (60 °C, fraction 1)	A34688 (B57379)	1,869,831	168,631	31.9 (31.4-32.4)	21.1 (20.7-21.6)	636 (98.8)	16,370	191	1 (21C, 9T)
SP11514	A50879 (B53695)	96,737	54,447	11.3 (10.8-11.9)	9.5 (9.0-10.1)	234 (99.0)	16,261	167	0
SP11515	A50880 (B53696)	437,843	186,034	7.4 (7.2-7.7)	5.7 (5.5-6.0)	785 (99.3)	16,313	115	0
SP11516	A50881 (B53697)	974,416	376,190	7.4 (7.2-7.6)	5.4 (5.2-5.5)	1687 (99.4)	16,332	100	0
SP11517	A50882 (B53698)	354,151	173,381	28.4 (28.0-28.9)	23.1 (22.6-23.6)	721 (99.2)	16,305	124	0
SP11518	A50883 (B53699)	475,975	49,041	30.6 (29.7-31.5)	23.9 (23.0-24.8)	200 (98.7)	16,217	209	1 (3C, 2T)
SP11519	A50884 (B53700)	708,390	298,668	21.2 (20.8-21.5)	15.9 (15.6-16.3)	1179 (99.5)	16,347	81	1 (3C, 2T)
SP11520	A50885 (B53701)	676,896	76,143	14.6 (14.0-15.1)	9.7 (9.2-10.2)	313 (98.1)	16,114	317	1 (12C, 5T)
SP11521	A50886 (B53702)	558,561	249,842	32 (31.6-32.4)	25.4 (24.9-25.8)	1,109 (99.3)	16,309	118	0
Extraction negative control	A50854 (B53694)	17,833	0	N/A	N/A	0	0	0	0

Supplementary table 6.2. List of ancient wapiti samples used in this study. Geographical origin and age of the wapiti bones used in this study. Calibration of dates was performed with IntCal20[12] and OxCal 4.4[13] software. All samples were provided by the Russian Academy of Sciences, Ekaterinburg, Russia (RASE).

MPI ID	Library ID	Catalog number	Museum	Region	Radiocarbon date, uncalibrated	Calibrated median and 95% range	C14 lab number/ Dating reference number	NCBI accession number
SP11514	A50879	1010/5738	RASE	Russia, Baislantash (Urals)	2,095±28	2,060; 2,150-1,950	OxA-22168	MG020564
SP11515	A50880	597/535	RASE	Russia, Zhukovskaya (Urals)	355±55	400; 510-300	OxA-22101	MG020563
SP11516	A50881	1294/13	RASE	Russia, Staroe Logovo (Urals)	1,761±28	1,650; 1,720-1,570	OxA-22169	MG020569
SP11517	A50882	1352/130	RASE	Russia, Birukova (Urals)	11,240±55	13,140; 13,290-13,080	OxA-22170	MG020566
SP11518	A50883	688/11	RASE	Russia, Krasnoselskaya (Urals)	1,825±26	1,720; 1,830-1,630	OxA-22104	MG020565
SP11519	A50884	1440/20	RASE	Russia, Sikiaz-Tamak (Urals)	12,135±60	14,030; 14,170-13,800	OxA-22171	MG020571
SP11520	A50885	1659/246	RASE	Russia, Kashinskoe (Urals)	368±25	430, 500-310	OxA-22100	MG020567
SP11521	A50886	798/2073	RASE	Russia, Shaitanskaya (Urals)	47,300±2900	50,470; >50,000-46,350	OxA-22102	MG020570

Supplementary table 6.3. Overview of log likelihoods for different clock models. The estimated log likelihoods for different tree and clock models using a path sampling approach.

Test-set	Clock model	Tree model	Marginal log likelihood
Mutation rate	Strict	Constant	-26282.36
	Strict	Exponential	-26286.71
	Relaxed log normal	Constant	-26346.45
	Relaxed log normal	Exponential	-26351.98
Tree building and molecular dating	Strict	Constant	-26416.59
	Strict	Bayesian skyline	-26387.30
	Relaxed log normal	Constant	-26415.85
	Relaxed log normal	Bayesian skyline	-26390.14

SI7: Denisova Cave pendant – human nuclear DNA analysis

Nuclear DNA processing and contamination estimates

The eleven libraries from the first, second and third 90°C phosphate fractions from DCP1 that were enriched for human nuclear DNA by hybridization capture (see Methods section and Supplementary information) were processed as described in Vernot et al (2021)[53]. Briefly, all sequences were mapped to a "third-allele" version of the hg19/GRCH37 reference genome[53] where each target SNP in the reference was replaced by a third allele that was neither the expected ancestral or derived allele. Unmapped sequences, sequences not overlapping the target sites and sequences shorter than 35 base pairs and with a mapping quality of less than 25 were removed. PCR duplicates were collapsed into single sequences using *bam-rmdup* (https://github.com/mpieva/biohazard-tools/tree/v0.2-knowngood). Kraken (version 1)[86] was then used to identify primate sequences. The primate classified sequences from each library were then evaluated for mammalian contamination using diagnostic positions where primates are known to carry derived alleles[53]. Using this approach, we determined that all libraries have less than 1% mammalian contamination (Data file S2).

Using AuthentiCT[56] we estimated contamination with present-day human DNA to 1.8% in the library prepared from the first 90 °C phosphate fraction, and to 0.1% for libraries prepared from the second and third 90 °C fractions (see Data file S2). Therefore, only the libraries from the second and third 90°C fractions were used for downstream analyses. Primate sequences from these ten libraries were merged, resulting in 336,429 SNPs covered by at least one sequence (or 145,590 SNPs when restricting to sequences from putatively deaminated DNA fragments, *i.e.* sequences with at least one C-to-T substitution within the first three or last three positions). Estimated modern human contamination amounts to 0.3% among all fragments for the merged dataset (see Data file S2), indicating that it is unnecessary to remove contaminant sequences by restricting analysis to deaminated fragments.

Data filtering for population genetic analyses

In order to determine how the DNA from the human individual isolated from DCP1 relates to DNA from previously published present-day and ancient humans, we performed a series of analyses in which we restricted the data to sequences of least 35 bp mapping to target positions with a mapping quality score of at least 25. Haploid genotypes were then obtained using *bam-caller* (https://github.com/bodkan/bam-caller, version: 0.2) which samples an allele from a randomly selected sequence at each target position. To limit the impact of cytosine deamination, T's in the first three and last three positions on forward strands and A's in the first three and last three positions on reverse strands were not sampled.

Principal Component Analysis (PCA)

We computed principal components (PCs) of 2,970 present-day humans from Africa, West Eurasia, the Americas, Central Asia and Siberia, and East Asia genotyped on 597,573 SNPs of the Affymetrix Human Origins array[87] using s*martpca*[88,89]. We then projected DCP1 and a set of 16 ancient modern populations (56 individuals) composed of Eurasian Palaeolithic individuals (>15 ka), ancient Native Americans, and ancient Baikal, Central Steppe, and Northeast Asian individuals onto the PCs defined by present-day humans (Supplementary table 7.1, Supplementary fig. 7.2). These individuals were selected due to either their temporal (Palaeolithic) or geographic proximity to DCP1 or previously identified relationships to other ancient individuals from the same temporal and geographic area as DCP1 (e.g. ancient Native Americans). In the PCA, DCP1 was found to fall within the variation of ancient west Eurasians and ancient north Eurasians.

We then constructed a second PCA that excluded Africans (Fig. 3C). In this PCA, DCP1 falls along the cline between Americans and West Eurasians, closest to the individuals in the population previously defined as Ancient North Eurasians (ANE), specifically the ~24-ka Ma'lta 1 and ~17-ka Afontova Gora 3, as well as West Siberian hunter gatherers.

f3-statistics

To calculate shared genetic drift between DCP1 and a selection of present-day and ancient human populations, we calculated *f3*-statistics using the set of SNPs within the "1240k" SNP panel and the R package *admixr* (version 0.7.1)[90]. For this statistic we used *f3(X,Y; Mbuti)*, where Mbuti is used as an outgroup to measure the genetic drift between populations *X* and *Y*. We used 124 different present-day populations from Simons Genome Diversity Project (SGDP)[91] as population *X* and calculated *f3*-statistics with and without restricting the DCP1 data (population *Y*) to deaminated fragments, thereby including 100,374 - 112,257 or 233,776-261,312 SNPs in the analyses, respectively. DCP1 showed the highest shared genetic drift with present-day Native American populations (Supplementary fig. 7.3, Supplementary table 7.2). This is consistent with DCP1 sharing some genetic ancestry with these populations.

In addition, we calculated the shared genetic drift between DCP1 and the selection of ancient modern humans described above (Supplementary fig. 7.4). For DCP1 all fragments were used for calculating the *f3*-statistic, resulting in 7,220 to 256,124 overlapping SNPs with different ancient humans. DCP1 shared the most drift with Ancient North Eurasians.

D-Statistics

We used D-statistics in order to directly evaluate the relationship of DCP1 to present-day populations and previously published ancient modern humans. For this analysis we used the "1240k" SNPs panel and calculated the D-statistics using the R package *admixr*, which was also used for the *f3*-statistics described above. Consistent with the PCA and *f3*-statistics, DCP1 was closer to present day non-Africans than Africans (Supplementary fig. 7.5), and closer to present-day Americans than other non-Africans (Supplementary fig. 7.6).

As DCP1 clustered with two other Ancient North Eurasians (ANE) (Mal'ta1 and Afontova Gora 3) in the PCA and *f3*-statistics, we tested these relationships in more depth. Computing *D(W, Mal'ta1; DCP1, Mbuti),* we found that, with the exception of another ANE individual (Afontova Gora 3), DCP1 was significantly closer to Mal'ta1 than to any other

ancient modern human we compared it to (W) (Extended data fig. 5B). When repeating the statistic with the positions of Mal'ta1 and DCP1 switched, *i.e. D(W, DCP1; Mal'ta1, Mbuti),* the same was observed for Mal'ta1 (Extended data fig. 5B), indicating that ancient human DNA from DCP1 originates from an Ancient North Eurasian. As ANEs are known to have contributed ancestry to ancient Americans and Siberians[92-95], we next explored if DCP1 had the same affinities to the ancient Siberians and ancient Americans as Mal'ta1 and Afontova Gora 3. When comparing both within and between different ancient Siberians and ancient Americans, DCP1 followed the same trends as the other two ANEs (Extended data fig. 5A and 5C, Supplementary figure 7.5, Data file S3). This further supports DCP1 belonging to or being closely related to ANEs.

Sex determination

The sex of ancient individuals can be determined genetically by comparing the relative amount of DNA originating from the X vs the autosomal chromosomes[96]. We previously applied this approach to show that several sediment samples containing Neandertal DNA originated primarily from one sex (3 male and 3 female[53]). This observation, combined with the fact that several of these also contained only a single mitochondrial haplotype, was taken as evidence that for many of these samples the majority of DNA originated from a single individual.

The probe-set used here for nuclear DNA capture (see Methods section and Supplementary information 4) is a sediment-specific probe-set based on the "1240k" SNP panel commonly used in ancient human genetics studies[69]. Unfortunately, only a small proportion of the probes in the 1240k panel overlap the X chromosome (0.38%), making a sex determination challenging. Therefore, we examined the shotgun data generated from the second 90°C phosphate fraction (library A34692, Data file S1). However, the use of shotgun data introduces the issue of faunal DNA potentially incorrectly mapping to the human genome. Sediment-specific probe-sets avoid this issue by targeting regions of high mammalian diversity, as described previously[53]. Therefore, we took an approach to simultaneously estimate sex and faunal mis-mapping.

Specifically, we selected shotgun reads that overlap approximately three million sites, which were previously ascertained to be informative for faunal mis-mapping[53]. These sites

are binned by the minimum distance from any non-mammalian genome ("burden"). Of the 272,775,081 reads generated, 55,503 unique adapter-trimmed reads with a length of 35 bp or more mapped of these sites in the human reference genome (hg19/GRCH37) with a map quality score of 25 or greater using BWA (version 0.5.10-evan.9-1-g44db244)[79] with 'ancient parameters' ('-n 0.01 –o 2 –l 16500')[80]. Using these reads we calculated the proportion of derived alleles in each bin (Supplementary fig. 7.1), thus arriving at an estimate of faunal mis-mapping per bin.

As expected, we observed substantial faunal mis-mapping for less diverse sites (Supplementary fig. 7.1, left of dotted line), but this mis-mapping was substantially reduced for sites with burden >= 8, with 98.4% of these reads estimated to originate from humans. We therefore selected the 20,526 reads overlapping these sites, and calculated X-autosome proportions. We did the same for a set of previously published sequence data from ancient hominin skeletal samples (Goyet Q56-1, Les Cottés Z4-1514, Mezmaiskaya 2, Spy 94a, Vindija 87[97], Mezmaiskaya 1[76]). We find that the X-autosome proportions in the pendant are consistent with known female samples, and approximately 2x those observed in known male samples (Fig. 3B). We performed the same analysis using only the 10,066 deaminated reads overlapping informative sites, 3,734 of which have burden >= 8, and reached the same results (Fig. 3B, Supplementary fig. 7.1).

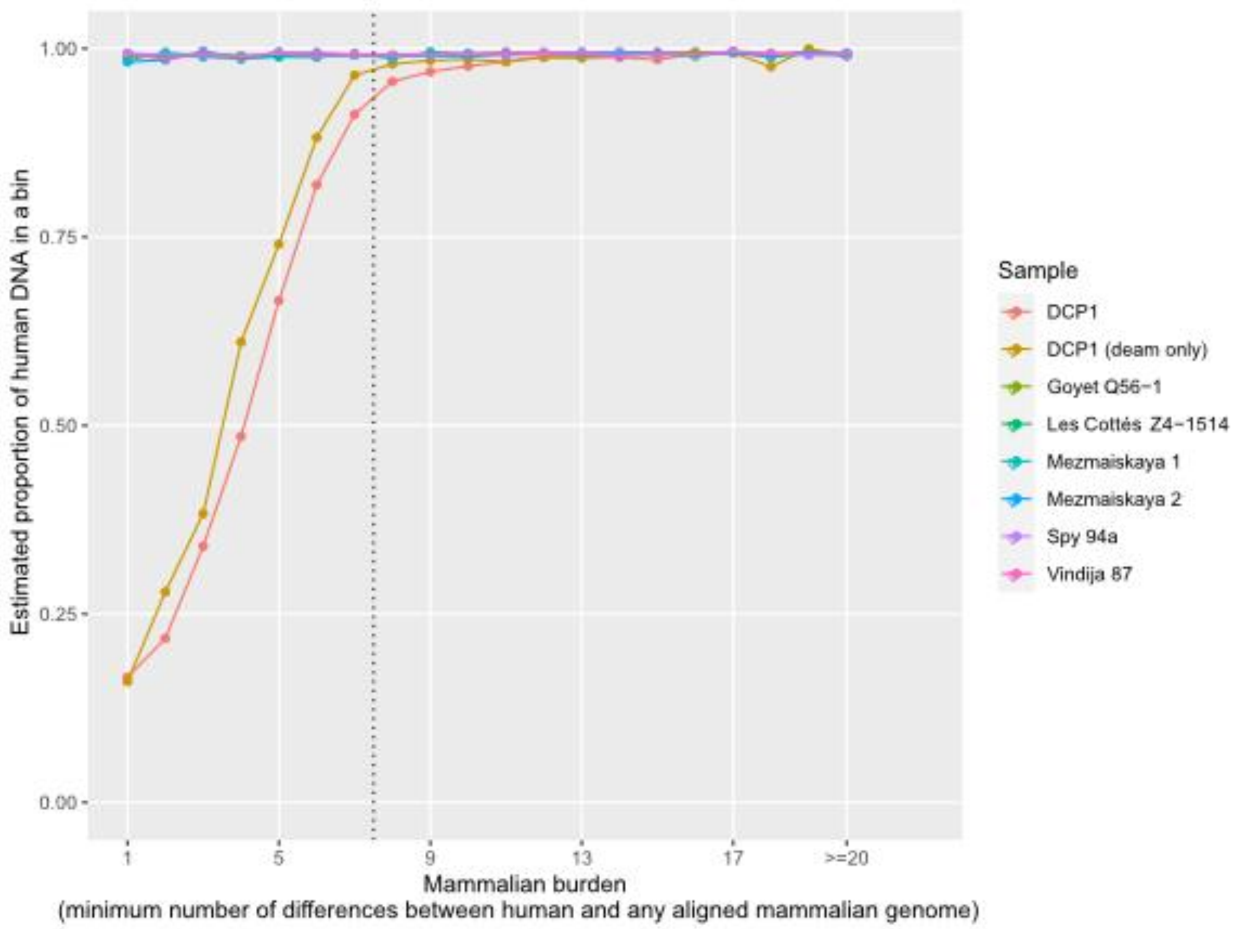

Supplementary figure 7.1. Estimation of faunal mis-mapping. Proportion of derived alleles at sites previously ascertained to be informative for faunal mis-mapping to the human genome[53]. Human DNA will have the derived allele, and non-human DNA will carry ancestral alleles. Data are shown for DCP1 (blue line) and for six previously published ancient hominin skeletal samples (other line colors). Sites are binned by "Mammalian burden," a measurement of the minimum divergence to the human genome from a set of 9 non-primate mammalian genomes. Faunal mis-mapping is substantially reduced for burdens >= 8 (vertical dotted line), and thus only these sites are considered for further analysis.

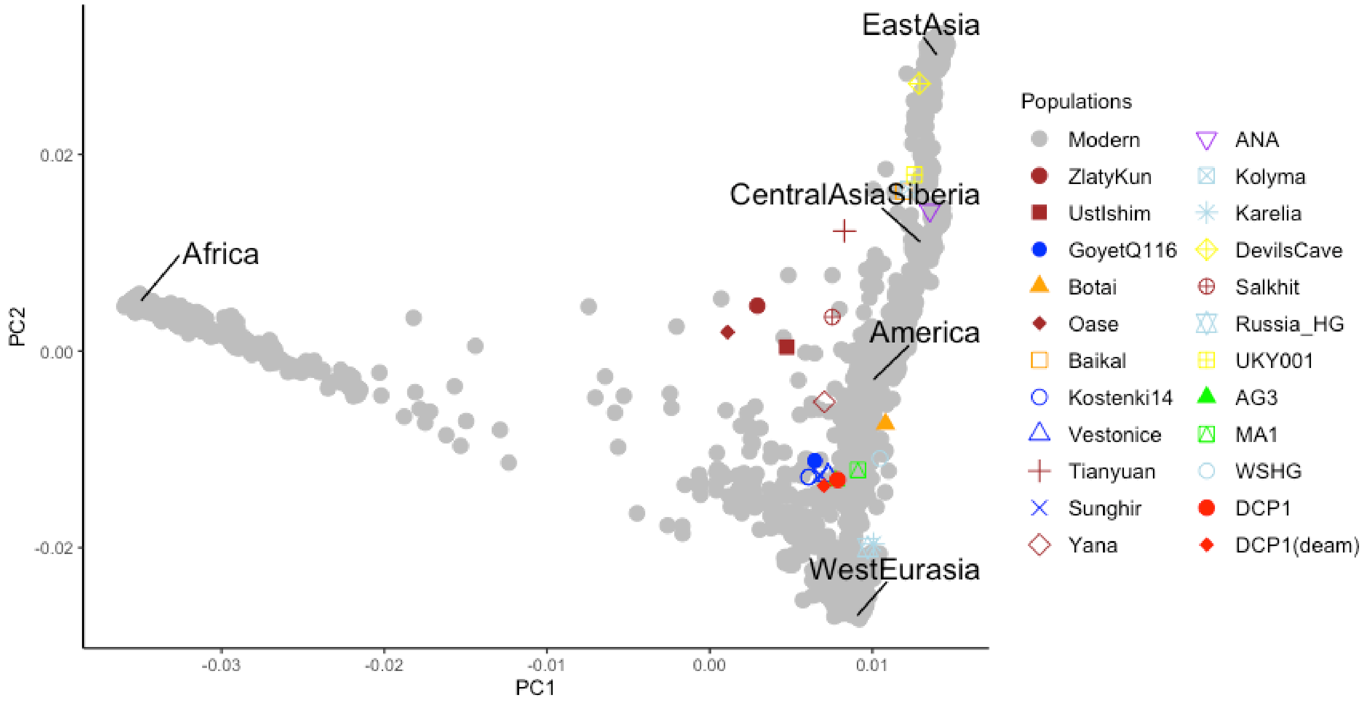

Supplementary figure 7.2. PCA of modern African and non-African human genomes with ancient human genomes projected on top. The modern individuals are colored in grey (see Supplementary table 7.1 for individuals in each ancient population). ANA = ancient Native Americans; Russia_HG = Russian hunter gatherers; UKY001 = Ust Kyakhta; AG3 =Afontova Gora 3; MA1 = Ma'lta 1; WSHG = West Siberian hunter gatherers.

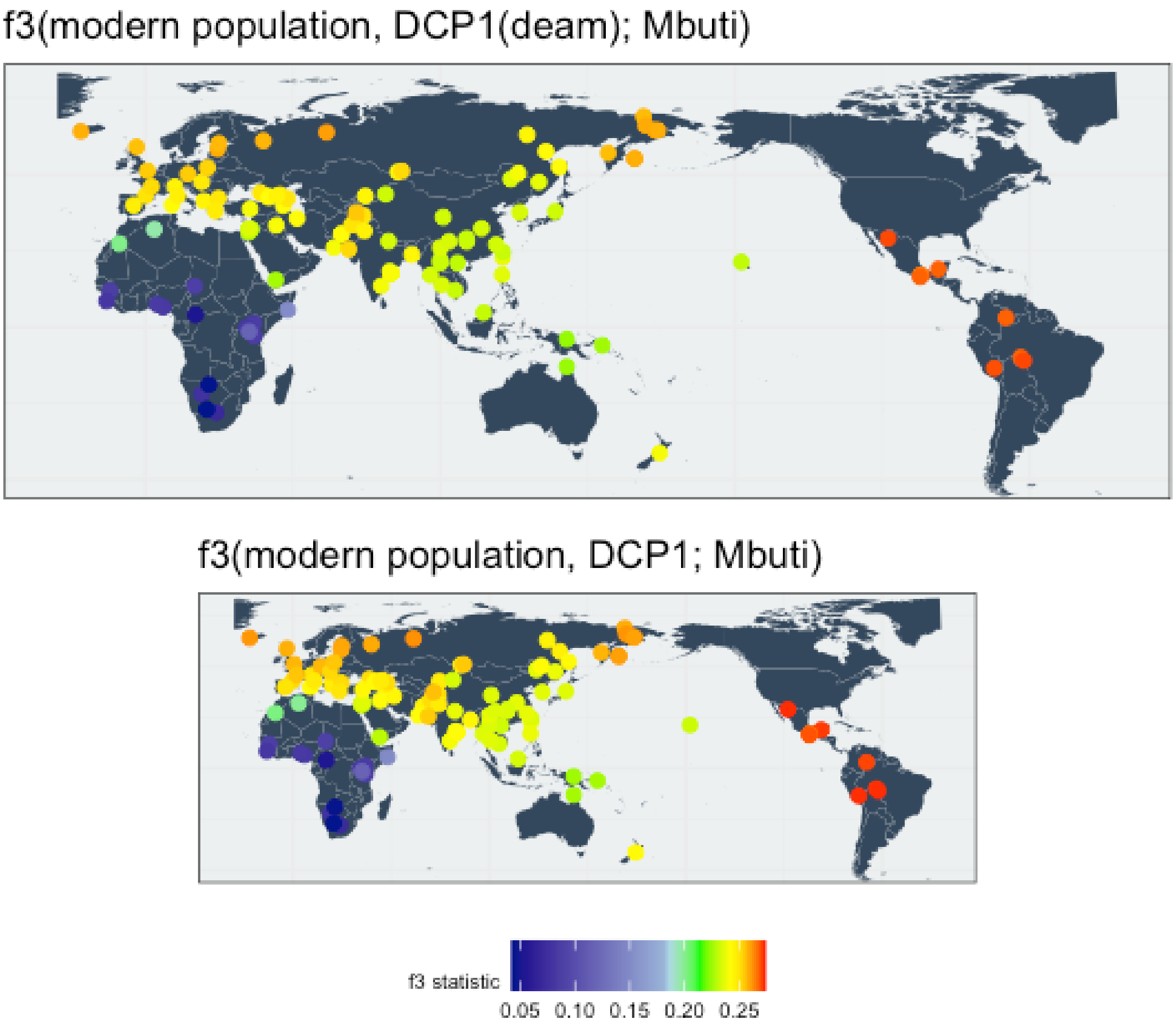

Supplementary figure 7.3. Shared genetic drift between the DCP1 and different present-day human populations. Each point represent a distinct statistic using the calculation $f_3(X,Y;\ Mbuti)$, where Mbuti serves as an outgroup, population *X* is a present-day human population and *Y* is either deaminated only fragments (top) or all (bottom) fragments from the Denisova Pendant. Warmer colors in the map[98] represent more shared genetic drift. Values for the f_3 statistics are reported in Supplementary table 7.2.

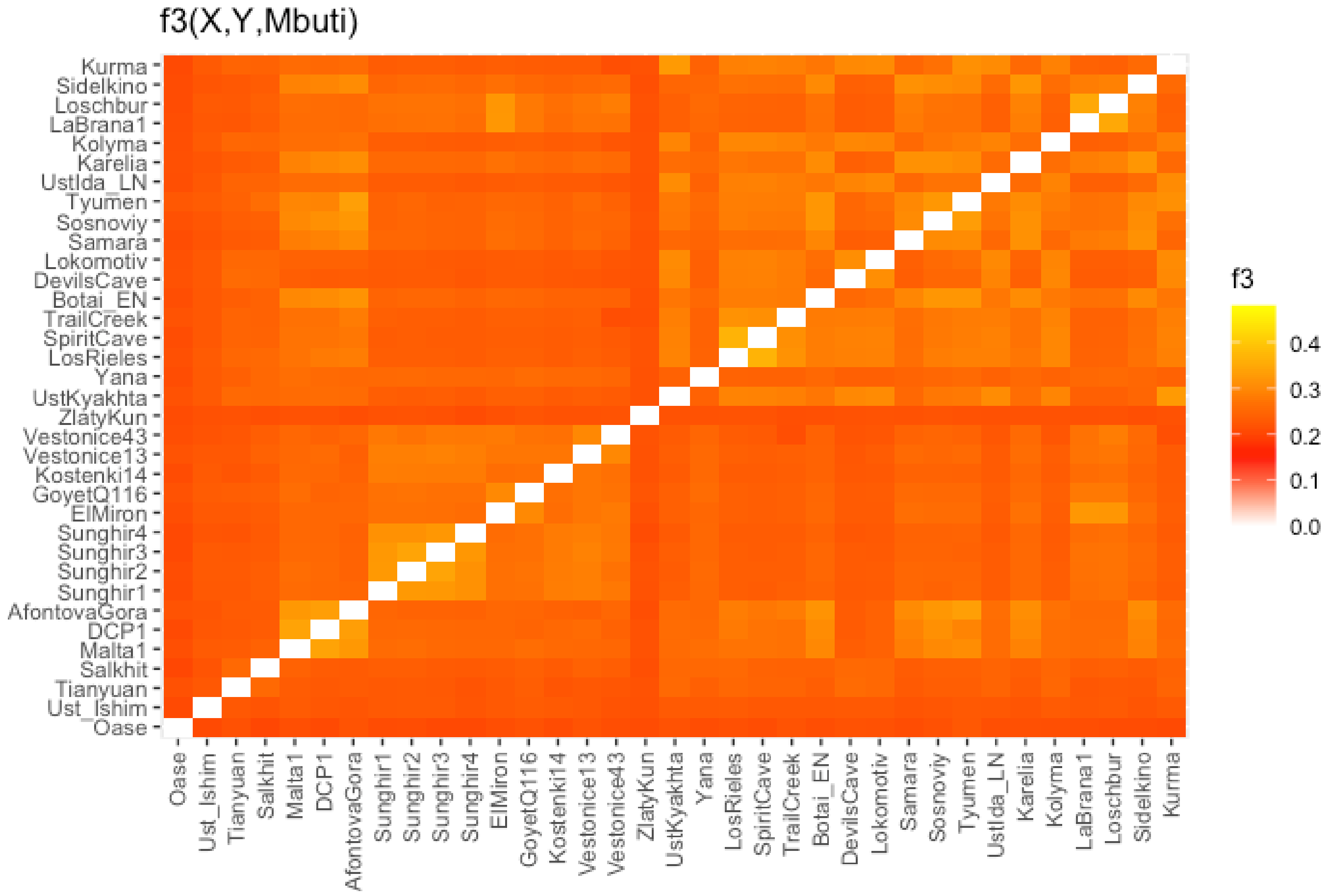

Supplementary figure 7.4. Heat map of f_3 statistics comparing a selection of ancient modern humans. Heat map of shared genetic drift between a selection of ancient humans (listed in the Supplementary table 7.1) as calculated with f_3 statistics using SNPs on the “1240k” array. Each grid point represents an individual statistic with yellow shades representing high amounts of shared genetic drift and red representing lower amounts. No deamination filter was applied for the DCP1 data.

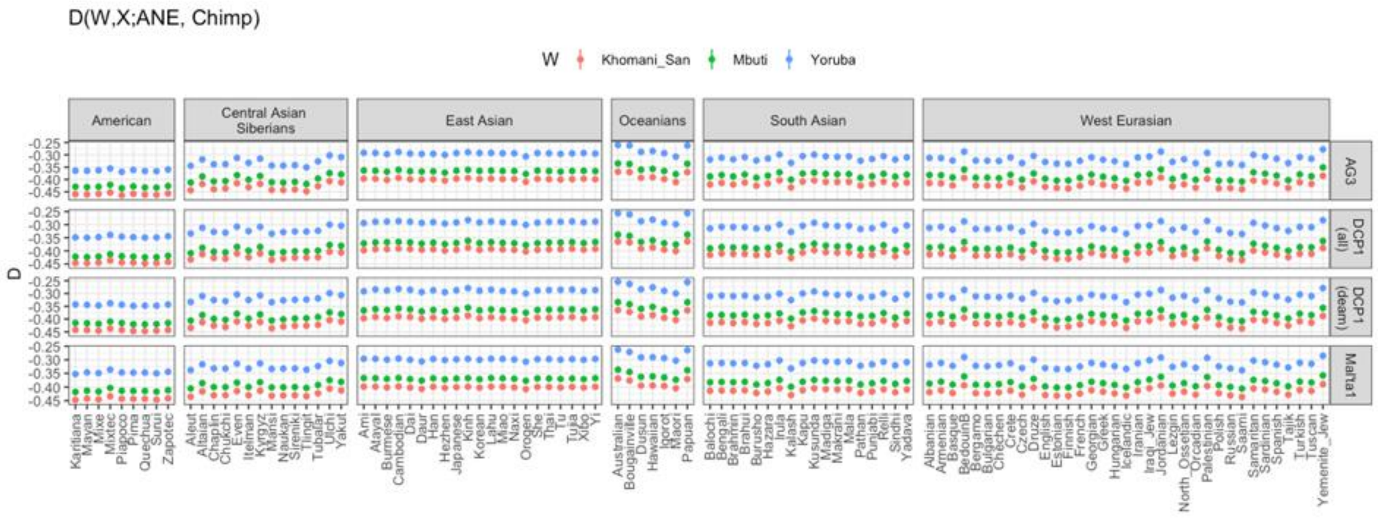

Supplementary figure 7.5. Genetic affinity of DCP1 and ANE individuals to present-day Africans and non-Africans/Americans calculated by D-statistics. Calculated D-statistics comparing the genetic affinity of DCP1 and ANE individuals to present-day Africans (W) versus non-Africans (X) (plotted on the x-axis; Data file S3). The calculation was performed using ADMIXTOOLS via *admixr* and using all fragments or deaminated fragments for DCP1. Each of the comparisons, reflected in the calculated D values, are plotted as circles on the y-axis. The corresponding error bars, plotted here as whiskers, represent one standard error (SE) as calculated by a Weighted Block Jackknife and a block size of 5 Mb across all autosomes (note that the standard errors are small and thus hardly visible in this plot; the individual standard error values for each of the comparisons are reported in Data file S3). All values had a significant Z-score (|Z| ≥ 3). The calculations are based on overlapping SNPs from the “1240k” SNP panel, with n(SNPs) = 139,962-326,182 per comparison. AG = Afontova Gora 3.

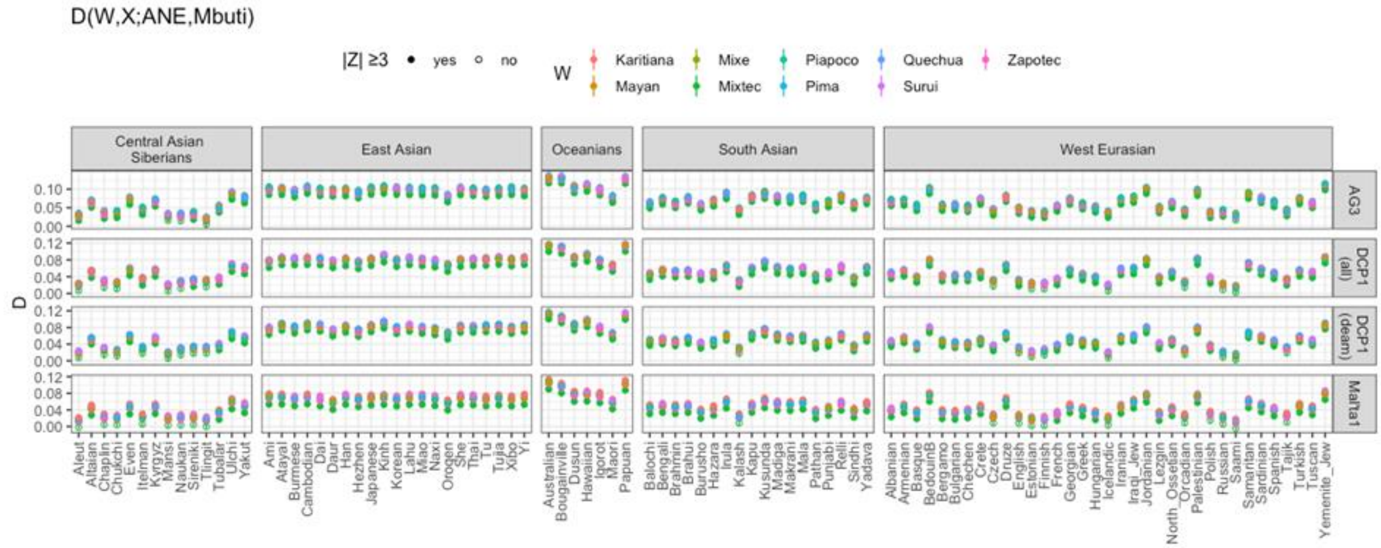

Supplementary figure 7.6. Genetic affinity of DCP1 and ANE individuals to present-day Americans and non-Africans/Americans calculated by D-statistics. Calculated D-statistics comparing the genetic affinity of DCP1 and ANE individuals to present-day Americans (W) versus non-Africans/Americans (X) (plotted on the x-axis; Data file S4). The calculation was performed using ADMIXTOOLS via admixr and using all fragments or deaminated fragments only for DCP1. Each of the comparisons, reflected in the calculated D values, are plotted as circles on the y-axis. The corresponding error bars, plotted here as whiskers, represent one standard error (SE) as calculated by a Weighted Block Jackknife and a block size of 5 Mb across all autosomes (note that the standard errors are small and thus hardly visible in this plot; the individual standard error values for each of the comparisons are reported in Data file S4). Filled-in circles indicate a significant Z-score or |Z| ≥ 3, and open circles indicate a non-significant Z-score, or |Z| < 3. The calculations are based on overlapping SNPs from the "1240k" SNP panel, with n(SNPs) = 142,414-331,740 per comparison. AG = Afontova Gora 3.

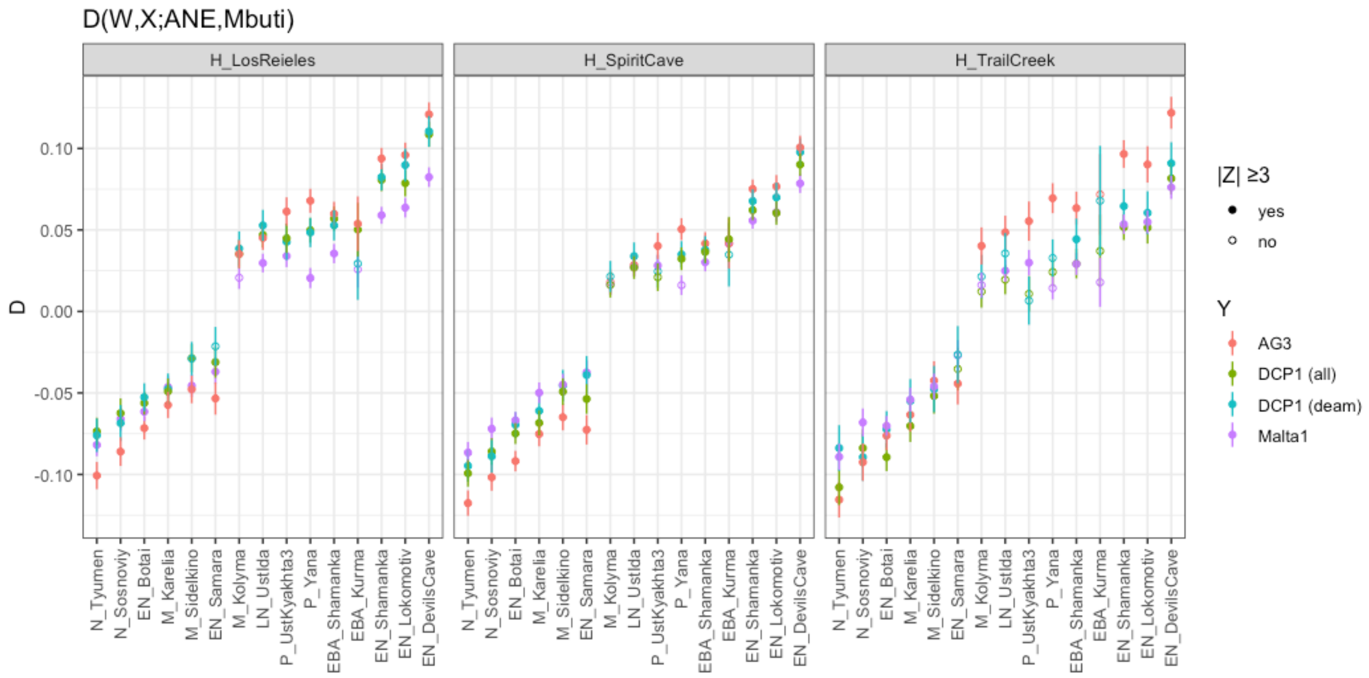

Supplementary figure 7.7. Genetic affinity of DCP1 and ANE individuals to ancient Siberians and ancient Americans calculated by D-statistics. Calculated D-statistics comparing the genetic affinity of DCP1 and ANE individuals (Y) to ancient Siberians (W, on the x-axis) and ancient Americans (X) (Data file S5). The calculation was performed using ADMIXTOOLS via admixr and using all fragments or deaminated fragments for DCP1. Each of the comparisons, reflected in the calculated D values, are plotted as circles. The corresponding error bars, plotted here as whiskers, represent one standard error (SE) as calculated by a Weighted Block Jackknife and a block size of 5 Mb across all autosomes. The calculations are based on overlapping SNPs from the “1240k” SNP panel, with n(SNPs) = 6,947-787,468 per comparison. Filled-in circles indicate a significant Z-score or |Z| ≥ 3, and open circles indicate a non-significant Z-score, or |Z| < 3. AG3 = Afontova Gora 3, EBA = Early Bronze Age, EN = Early Neolithic, LN = Late Neolithic, M = Mesolithic, N = Neolithic, P = Palaeolithic, H = Holocene.

Supplementary table 7.1. List of individuals used in population genetic analyses.

Previously published ancient humans included in population analyses.

Individual/Sample ID	Site	Geographical Region	Group	Approximate Age (years)	Reference
Botai 14	Botai	Central Steppe	Botai	Copper Age (5,400)	Damgaard et al., 2018[99]
Botai 15	Botai	Central Steppe	Botai	Copper Age (5,400)	Damgaard et al., 2018[99]
Botai 2016	Botai	Central Steppe	Botai	Copper Age (5,400)	Damgaard et al., 2018[99]
DA334	Shamanka_EBA	Cis-Baikal	Baikal	Early Bronze Age (4,000-3,800)	Damgaard et al., 2018[99]
DA335	Shamanka_EBA	Cis-Baikal	Baikal	Early Bronze Age (4,000-3,800)	Damgaard et al., 2018[99]
DA336	Shamanka_EBA	Cis-Baikal	Baikal	Early Bronze Age (4,000-3,800)	Damgaard et al., 2018[99]
DA337	Shamanka_EBA	Cis-Baikal	Baikal	Early Bronze Age (4,000-3,800)	Damgaard et al., 2018[99]
DA338	Shamanka_EBA	Cis-Baikal	Baikal	Early Bronze Age (4,000-3,800)	Damgaard et al., 2018[99]
DA339	Shamanka_EBA	Cis-Baikal	Baikal	Early Bronze Age (4,000-3,800)	Damgaard et al., 2018[99]
DA360	Kurma	Cis-Baikal	Baikal	Early Bronze Age (4,100)	Damgaard et al., 2018[99]
DA340	Lokomotiv	Cis-Baikal	Baikal	Early Neolithic (6,700)	Damgaard et al., 2018[99]
DA341	Lokomotiv	Cis-Baikal	Baikal	Early Neolithic (6,700)	Damgaard et al., 2018[99]
DA359	Lokomotiv	Cis-Baikal	Baikal	Early Neolithic (6,700)	Damgaard et al., 2018[99]
I0124	Samara	Russia	Eastern Hunter Gatherer	Early Neolithic (7,000)	Damgaard et al., 2018[99]
DA245	Shamanka	Cis-Baikal	Baikal	Early Neolithic (7,200-6,200)	Damgaard et al., 2018[99]
DA246	Shamanka	Cis-Baikal	Baikal	Early Neolithic (7,200-6,200)	Damgaard et al., 2018[99]
DA247	Shamanka	Cis-Baikal	Baikal	Early Neolithic (7,200-6,200)	Damgaard et al., 2018[99]
DA248	Shamanka	Cis-Baikal	Baikal	Early Neolithic (7,200-6,200)	Damgaard et al., 2018[99]
DA249	Shamanka	Cis-Baikal	Baikal	Early Neolithic (7,200-6,200)	Damgaard et al., 2018[99]
DA250	Shamanka	Cis-Baikal	Baikal	Early Neolithic (7,200-6,200)	Damgaard et al., 2018[99]
DA251	Shamanka	Cis-Baikal	Baikal	Early Neolithic (7,200-6,200)	Damgaard et al., 2018[99]
DA252	Shamanka	Cis-Baikal	Baikal	Early Neolithic (7,200-6,200)	Damgaard et al., 2018[99]
DA362	Shamanka	Cis-Baikal	Baikal	Early Neolithic (7,200-6,200)	Damgaard et al., 2018[99]
Devil's Gate	Devil's Cave	East Asia	Northeast Asian	Early Neolithic (7,700)	Sikora et al., 2019[100]
I11974	LosRieles	Chile	Ancient Native American	Holocene (11,000)	Posth et al., 2018[94]
Spirit Cave	SpiritCave	Nevada	Ancient Native American	Holocene (11,000)	Moreno-Mayar et al., 2018[92]
Trail Creek	TrailCreek	Alaska	Ancient Native American	Holocene (9,000)	Moreno-Mayar et al., 2018[92]
DA342	Ust'Ida	Cis-Baikal	Baikal	Late Neolithic (5,000)	Damgaard et al., 2018[99]
DA344	Ust'Ida	Cis-Baikal	Baikal	Late Neolithic (5,000)	Damgaard et al., 2018[99]
DA345	Ust'Ida	Cis-Baikal	Baikal	Late Neolithic (5,000)	Damgaard et al., 2018[99]
DA355	Ust'Ida	Cis-Baikal	Baikal	Late Neolithic (5,000)	Damgaard et al., 2018[99]
Sidelkino	Sidelkino	Cis-Baikal	Baikal	Mesolithic (11,000)	Damgaard et al., 2018[99]
Karelia	Karelia	Russia	Karelia	Mesolithic (8,000)	Mallick et al., 2016[91]
Kolyma 1	Duvanni Yar	Beringia	Ancient Palaeo Siberian	Mesolithic (9,800)	Sikora et al., 2019[91]
I1958	Tyumen Oblast	Siberia	West Siberian Hunter Gatherer	Neolithic (5,000)	Narasimhan et al., 2019[93]
Sosnoviy	Sosnoviy Ostrov	Siberia	West Siberian Hunter Gatherer	Neolithic (5,000)	Narasimhan et al., 2019[93]
I1960	Tyumen Oblast	Siberia	West Siberian Hunter Gatherer	Neolithic (6,000)	Narasimhan et al., 2019[93]
UKY001	Ust Kyakhta 3	Cis-Baikal	Ust Kyakhta	Palelothic (14,000)	Yu et al., 2020[95]
Afontova Gora 3	Afontova Gora	south central Siberia	Ancient North Eurasian	Palaeolithic (15,000)	Fu et al., 2016[101]
Ma'lta 1	Ma'lta	Cis-Baikal	Ancient North Eurasian	Palaeolithic (24,000)	Raghavan et al., 2014[102]
Yana 1	Yana RHS	Siberia	Yana	Palaeolithic (28,000)	Sikora et al., 2019[100]
Yana 2	Yana RHS	Siberia	Yana	Palaeolithic (28,000)	Sikora et al., 2019[100]
Vestonice 13	Vestonice	Czech Republic	Dolni Vestonice	Palaeolithic (30,000)	Fu et al., 2016[101]
Vestonice 43	Vestonice	Czech Republic	Dolni Vestonice	Palaeolithic (30,000)	Fu et al., 2016[101]
Vestonice 16	Vestonice	Czech Republic	Dolni Vestonice	Palaeolithic (30,000)	Fu et al., 2016[101]
Sunghir 1	Sunghir	Russia	Sunghir	Palaeolithic (34,000)	Sikora et al., 2017[103]
Sunghir 2	Sunghir	Russia	Sunghir	Palaeolithic (34,000)	Sikora et al., 2017[103]
Sunghir 3	Sunghir	Russia	Sunghir	Palaeolithic (34,000)	Sikora et al., 2017[103]
Sunghir 4	Sunghir	Russia	Sunghir	Palaeolithic (34,000)	Sikora et al., 2017[103]
Salkhit	Salkhit Valley	Mongolia	Salkhit	Palaeolithic (34,000)	Massilani et al., 2020[104]
GoyetQ116	Goyet	Belgium	GoyetQ116	Palaeolithic (35,000)	Fu et al., 2016[101]
Kostenki 14	Kostenki	Russia	Kostenki14	Palaeolithic (37,000)	Fu et al., 2016[101]
Oase1	Oase	Romania	Oase	Palaeolithic (40,000)	Jones et al., 2015[105]
Tianyuan	Tianyuan	China	Tianyuan	Palaeolithic (40,000)	Fu et al., 2013[71]
Ust'Ishim	Ust'Ishim	Siberia	Ust'Ishim	Palaeolithic (45,000)	Fu et al., 2014[70]
ZlatyKun	ZlatyKun	Czech Republic	ZlatyKun	Palaeolithic (>35,000)	Prufer et al., 2021[76]

Supplementary table 7.2. Calculated f_3-statistics using f_3*(modern population, Denisova pendant; Mbuti)*. This statistic was calculated with ADMIXTOOLS[87] via the R package *admixr*[90] with standard errors (SE) calculated using a Weighted Block Jackknife[87,106] and a block size of 5 Mb across all autosomes of the "1240k" SNP panel. The number of overlapping SNPs between the individuals or populations included in each comparison is reported in "# SNPs" column. The modern populations used (columns A and C) are from the SGDP[91] and separate statistics were calculated for both all and only DNA fragments containing putative deamination from the Denisova Cave pendant (column B). A visual representation of these results is shown in Supplementary fig. 7.3.

A	B	C	f3	SE	Zscore	# SNPs
Abkhasian	DenPen (all)	Mbuti	0.243766	0.003754	64.938	107,090
Abkhasian	DenPen (deam)	Mbuti	0.246481	0.003412	72.23	249,413
Adygei	DenPen (all)	Mbuti	0.248936	0.003738	66.604	107,213
Adygei	DenPen (deam)	Mbuti	0.251511	0.003393	74.116	249,719
Albanian	DenPen (all)	Mbuti	0.24499	0.003889	63.003	102,172
Albanian	DenPen (deam)	Mbuti	0.247299	0.003512	70.415	237,771
Aleut	DenPen (all)	Mbuti	0.260204	0.003696	70.41	109,702
Aleut	DenPen (deam)	Mbuti	0.262348	0.003382	77.564	255,512
Altaian	DenPen (all)	Mbuti	0.24365	0.004196	58.068	101,889
Altaian	DenPen (deam)	Mbuti	0.247001	0.003724	66.33	237,165
Ami	DenPen (all)	Mbuti	0.237243	0.00395	60.065	105,660
Ami	DenPen (deam)	Mbuti	0.238844	0.003487	68.496	246,085
Armenian	DenPen (all)	Mbuti	0.242236	0.003652	66.335	107,230
Armenian	DenPen (deam)	Mbuti	0.245035	0.003325	73.703	249,790
Atayal	DenPen (all)	Mbuti	0.231652	0.004171	55.543	101,500
Atayal	DenPen (deam)	Mbuti	0.234897	0.003784	62.074	236,183
Australian	DenPen (all)	Mbuti	0.224387	0.00399	56.243	104,873
Australian	DenPen (deam)	Mbuti	0.226313	0.003524	64.216	244,411
Balochi	DenPen (all)	Mbuti	0.246675	0.003838	64.276	106,802
Balochi	DenPen (deam)	Mbuti	0.249646	0.003396	73.514	248,689
BantuHerero	DenPen (all)	Mbuti	0.07706	0.002568	30.003	104,549
BantuHerero	DenPen (deam)	Mbuti	0.07727	0.002189	35.306	243,413
BantuKenya	DenPen (all)	Mbuti	0.085992	0.002589	33.219	104,606
BantuKenya	DenPen (deam)	Mbuti	0.086034	0.002311	37.236	243,384
BantuTswana	DenPen (all)	Mbuti	0.070477	0.002379	29.63	104,837
BantuTswana	DenPen (deam)	Mbuti	0.07148	0.002045	34.945	244,199
Basque	DenPen (all)	Mbuti	0.249618	0.003833	65.12	107,135
Basque	DenPen (deam)	Mbuti	0.252079	0.003468	72.686	249,487
BedouinB	DenPen (all)	Mbuti	0.228855	0.00375	61.027	106,394
BedouinB	DenPen (deam)	Mbuti	0.231893	0.003273	70.849	247,670
Bengali	DenPen (all)	Mbuti	0.24407	0.00375	65.078	107,023
Bengali	DenPen (deam)	Mbuti	0.244485	0.003359	72.794	249,057
Bergamo	DenPen (all)	Mbuti	0.245494	0.003993	61.474	101,957
Bergamo	DenPen (deam)	Mbuti	0.249722	0.003612	69.131	237,599
Biaka	DenPen (all)	Mbuti	0.05153	0.002267	22.731	103,140
Biaka	DenPen (deam)	Mbuti	0.052307	0.001928	27.131	240,069
Bougainville	DenPen (all)	Mbuti	0.225601	0.003808	59.251	104,811
Bougainville	DenPen (deam)	Mbuti	0.226322	0.00348	65.027	244,275
Brahmin	DenPen (all)	Mbuti	0.245576	0.003633	67.6	107,005
Brahmin	DenPen (deam)	Mbuti	0.24606	0.003224	76.331	249,280
Brahui	DenPen (all)	Mbuti	0.241934	0.003764	64.28	107,022
Brahui	DenPen (deam)	Mbuti	0.244883	0.003355	72.996	249,137
Bulgarian	DenPen (all)	Mbuti	0.248733	0.004032	61.684	101,736
Bulgarian	DenPen (deam)	Mbuti	0.251669	0.003647	69.005	236,991
Burmese	DenPen (all)	Mbuti	0.233704	0.003823	61.127	106,715
Burmese	DenPen (deam)	Mbuti	0.235336	0.00336	70.049	248,476
Burusho	DenPen (all)	Mbuti	0.249791	0.003735	66.885	107,092
Burusho	DenPen (deam)	Mbuti	0.250499	0.003389	73.925	249,581
Cambodian	DenPen (all)	Mbuti	0.231748	0.003801	60.968	106,533
Cambodian	DenPen (deam)	Mbuti	0.234322	0.003429	68.334	247,909
Chechen	DenPen (all)	Mbuti	0.247754	0.004187	59.174	101,867
Chechen	DenPen (deam)	Mbuti	0.24978	0.003703	67.451	237,423
Chukchi	DenPen (all)	Mbuti	0.256533	0.004121	62.254	102,208
Chukchi	DenPen (deam)	Mbuti	0.258934	0.003703	69.93	238,215
Crete	DenPen (all)	Mbuti	0.244079	0.003739	65.285	107,196
Crete	DenPen (deam)	Mbuti	0.246816	0.003313	74.507	249,641
Czech	DenPen (all)	Mbuti	0.254071	0.004168	60.95	102,151
Czech	DenPen (deam)	Mbuti	0.257248	0.003714	69.26	237,686
Dai	DenPen (all)	Mbuti	0.23201	0.003756	61.766	110,157
Dai	DenPen (deam)	Mbuti	0.234938	0.003381	69.482	256,756
Daur	DenPen (all)	Mbuti	0.235428	0.004116	57.201	101,855
Daur	DenPen (deam)	Mbuti	0.236851	0.003686	64.256	237,062

A	B	C	f3	SE	Zscore	# SNPs
Khomani_San	DenPen (all)	Mbuti	0.042091	0.002277	18.488	103,531
Khomani_San	DenPen (deam)	Mbuti	0.043351	0.001924	22.53	240,854
Khonda_Dora	DenPen (all)	Mbuti	0.237186	0.004125	57.502	101,625
Khonda_Dora	DenPen (deam)	Mbuti	0.238911	0.003575	66.825	236,772
Kinh	DenPen (all)	Mbuti	0.230437	0.003821	60.303	106,215
Kinh	DenPen (deam)	Mbuti	0.232694	0.003381	68.816	247,413
Korean	DenPen (all)	Mbuti	0.234275	0.003856	60.759	106,356
Korean	DenPen (deam)	Mbuti	0.236301	0.003437	68.745	247,509
Kusunda	DenPen (all)	Mbuti	0.234068	0.00383	61.115	105,493
Kusunda	DenPen (deam)	Mbuti	0.237337	0.00348	68.205	245,747
Kyrgyz	DenPen (all)	Mbuti	0.244775	0.003714	65.909	107,188
Kyrgyz	DenPen (deam)	Mbuti	0.247215	0.003355	73.685	249,579
Lahu	DenPen (all)	Mbuti	0.232315	0.003878	59.905	105,808
Lahu	DenPen (deam)	Mbuti	0.234908	0.003483	67.435	246,588
Lezgin	DenPen (all)	Mbuti	0.249508	0.003804	65.597	107,158
Lezgin	DenPen (deam)	Mbuti	0.251608	0.003401	73.988	249,499
Luhya	DenPen (all)	Mbuti	0.085876	0.002707	31.724	104,504
Luhya	DenPen (deam)	Mbuti	0.08516	0.002395	35.551	243,507
Luo	DenPen (all)	Mbuti	0.082959	0.00267	31.066	104,731
Luo	DenPen (deam)	Mbuti	0.083319	0.002284	36.474	243,653
Madiga	DenPen (all)	Mbuti	0.241762	0.003702	65.306	106,826
Madiga	DenPen (deam)	Mbuti	0.24361	0.003315	73.496	248,778
Makrani	DenPen (all)	Mbuti	0.239892	0.003664	65.481	106,742
Makrani	DenPen (deam)	Mbuti	0.241952	0.003307	73.171	248,636
Mala	DenPen (all)	Mbuti	0.239476	0.003656	65.503	106,783
Mala	DenPen (deam)	Mbuti	0.242044	0.003184	76.008	248,762
Mandenka	DenPen (all)	Mbuti	0.087151	0.002552	34.155	108,189
Mandenka	DenPen (deam)	Mbuti	0.087496	0.002214	39.523	252,064
Mansi	DenPen (all)	Mbuti	0.260863	0.003894	66.999	106,773
Mansi	DenPen (deam)	Mbuti	0.26281	0.003482	75.47	248,751
Maori	DenPen (all)	Mbuti	0.239926	0.004182	57.369	102,252
Maori	DenPen (deam)	Mbuti	0.243007	0.003668	66.253	238,187
Masai	DenPen (all)	Mbuti	0.116935	0.002734	42.768	105,213
Masai	DenPen (deam)	Mbuti	0.11727	0.002391	49.054	245,045
Mayan	DenPen (all)	Mbuti	0.268277	0.004075	65.827	104,604
Mayan	DenPen (deam)	Mbuti	0.271754	0.003695	73.548	243,741
Mende	DenPen (all)	Mbuti	0.08095	0.002596	31.18	104,736
Mende	DenPen (deam)	Mbuti	0.080481	0.002227	36.14	243,876
Miao	DenPen (all)	Mbuti	0.233684	0.003901	59.904	106,157
Miao	DenPen (deam)	Mbuti	0.234903	0.003381	69.478	247,127
Mixe	DenPen (all)	Mbuti	0.268745	0.004028	66.718	106,018
Mixe	DenPen (deam)	Mbuti	0.270634	0.003673	73.675	247,107
Mixtec	DenPen (all)	Mbuti	0.264317	0.004003	66.037	105,486
Mixtec	DenPen (deam)	Mbuti	0.265698	0.003569	74.45	246,008
Mozabite	DenPen (all)	Mbuti	0.195856	0.003491	56.098	106,698
Mozabite	DenPen (deam)	Mbuti	0.198956	0.003094	64.307	248,764
Naxi	DenPen (all)	Mbuti	0.23513	0.003662	64.204	108,847
Naxi	DenPen (deam)	Mbuti	0.237117	0.003323	7	253,534
North_Ossetian	DenPen (all)	Mbuti	0.245365	0.003785	64.825	107,196
North_Ossetian	DenPen (deam)	Mbuti	0.247245	0.003333	74.176	249,944
Orcadian	DenPen (all)	Mbuti	0.255902	0.003843	66.585	107,427
Orcadian	DenPen (deam)	Mbuti	0.258451	0.003457	74.753	249,985
Oroqen	DenPen (all)	Mbuti	0.239736	0.003924	61.097	106,022
Oroqen	DenPen (deam)	Mbuti	0.24237	0.003514	68.971	246,785
Palestinian	DenPen (all)	Mbuti	0.229142	0.003623	63.251	110,327
Palestinian	DenPen (deam)	Mbuti	0.230177	0.00326	70.61	257,008
Papuan	DenPen (all)	Mbuti	0.2242	0.003711	60.409	111,775
Papuan	DenPen (deam)	Mbuti	0.225326	0.003384	66.592	260,331
Pathan	DenPen (all)	Mbuti	0.249832	0.003854	64.828	107,076
Pathan	DenPen (deam)	Mbuti	0.251477	0.003414	73.658	249,592
Piapoco	DenPen (all)	Mbuti	0.268356	0.004136	64.881	104,253
Piapoco	DenPen (deam)	Mbuti	0.270992	0.003726	72.721	242,894

Supplementary table 7.2. (cont.): **Calculated *f_3*-statistics using *f_3(modern population, Denisova pendant; Mbuti).***

A	B	C	f3	stderr	Zscore	# SNPs	A	B	C	f3	stderr	Zscore	# SNPs
Druze	DenPen (all)	Mbuti	0.238	0.004	64.83	107,134	Pima	DenPen (all)	Mbuti	0.271	0.004	65.07	104,165
Druze	DenPen (deam)	Mbuti	0.239	0.003	73.86	249,437	Pima	DenPen (deam)	Mbuti	0.273	0.004	73.04	242,625
Dusun	DenPen (all)	Mbuti	0.231	0.004	59.53	105,723	Polish	DenPen (all)	Mbuti	0.253	0.004	63.5	102,149
Dusun	DenPen (deam)	Mbuti	0.234	0.003	67.62	246,241	Polish	DenPen (deam)	Mbuti	0.255	0.004	71.8	237,690
English	DenPen (all)	Mbuti	0.254	0.004	64.46	107,168	Punjabi	DenPen (all)	Mbuti	0.246	0.004	67.86	112,257
English	DenPen (deam)	Mbuti	0.257	0.003	73.75	249,688	Punjabi	DenPen (deam)	Mbuti	0.248	0.003	76.76	261,312
Esan	DenPen (all)	Mbuti	0.084	0.003	32.04	104,596	Quechua	DenPen (all)	Mbuti	0.27	0.004	66.32	104,477
Esan	DenPen (deam)	Mbuti	0.083	0.002	36.5	243,784	Quechua	DenPen (deam)	Mbuti	0.273	0.004	74.22	243,749
Chaplin	DenPen (all)	Mbuti	0.261	0.004	58.47	101,293	Relli	DenPen (all)	Mbuti	0.239	0.004	65.79	106,966
Chaplin	DenPen (deam)	Mbuti	0.262	0.004	66.83	235,846	Relli	DenPen (deam)	Mbuti	0.241	0.003	73.58	249,072
Naukan	DenPen (all)	Mbuti	0.259	0.004	63.41	104,941	Russian	DenPen (all)	Mbuti	0.257	0.004	67.11	107,223
Naukan	DenPen (deam)	Mbuti	0.262	0.004	71.88	244,696	Russian	DenPen (deam)	Mbuti	0.259	0.003	75.18	249,652
Sireniki	DenPen (all)	Mbuti	0.259	0.004	64.12	105,299	Saami	DenPen (all)	Mbuti	0.263	0.004	66.32	106,673
Sireniki	DenPen (deam)	Mbuti	0.26	0.004	72.35	245,493	Saami	DenPen (deam)	Mbuti	0.265	0.004	75.27	248,548
Estonian	DenPen (all)	Mbuti	0.258	0.004	66.29	107,255	Saharawi	DenPen (all)	Mbuti	0.201	0.003	57.59	107,112
Estonian	DenPen (deam)	Mbuti	0.26	0.003	74.49	249,750	Saharawi	DenPen (deam)	Mbuti	0.202	0.003	64.41	249,317
Even	DenPen (all)	Mbuti	0.244	0.004	63.98	108,635	Samaritan	DenPen (all)	Mbuti	0.235	0.004	57.61	101,135
Even	DenPen (deam)	Mbuti	0.246	0.003	71.69	253,227	Samaritan	DenPen (deam)	Mbuti	0.236	0.004	64.62	235,496
Finnish	DenPen (all)	Mbuti	0.257	0.004	69.54	110,345	Sardinian	DenPen (all)	Mbuti	0.241	0.004	65.35	109,998
Finnish	DenPen (deam)	Mbuti	0.26	0.003	77.97	257,022	Sardinian	DenPen (deam)	Mbuti	0.243	0.003	73.15	256,266
French	DenPen (all)	Mbuti	0.252	0.004	66.71	110,464	She	DenPen (all)	Mbuti	0.233	0.004	60.25	105,884
French	DenPen (deam)	Mbuti	0.255	0.003	76.71	257,089	She	DenPen (deam)	Mbuti	0.237	0.003	67.94	246,460
Gambian	DenPen (all)	Mbuti	0.088	0.003	33.19	104,912	Sindhi	DenPen (all)	Mbuti	0.252	0.004	61.08	101,471
Gambian	DenPen (deam)	Mbuti	0.09	0.002	37.27	244,191	Sindhi	DenPen (deam)	Mbuti	0.254	0.004	69.52	236,456
Georgian	DenPen (all)	Mbuti	0.242	0.004	64.89	107,183	Somali	DenPen (all)	Mbuti	0.151	0.003	43.09	100,374
Georgian	DenPen (deam)	Mbuti	0.243	0.003	71.64	249,467	Somali	DenPen (deam)	Mbuti	0.15	0.003	48.64	233,776
Greek	DenPen (all)	Mbuti	0.247	0.004	65.38	107,267	Spanish	DenPen (all)	Mbuti	0.247	0.004	63.8	107,156
Greek	DenPen (deam)	Mbuti	0.249	0.003	73.25	249,636	Spanish	DenPen (deam)	Mbuti	0.25	0.003	72.48	249,719
Han	DenPen (all)	Mbuti	0.234	0.004	61.8	108,777	Surui	DenPen (all)	Mbuti	0.271	0.004	62.49	103,083
Han	DenPen (deam)	Mbuti	0.237	0.003	69.72	253,386	Surui	DenPen (deam)	Mbuti	0.273	0.004	70.65	240,243
Hawaiian	DenPen (all)	Mbuti	0.231	0.004	56.76	101,649	Tajik	DenPen (all)	Mbuti	0.255	0.004	65.46	106,991
Hawaiian	DenPen (deam)	Mbuti	0.232	0.004	63.94	236,701	Tajik	DenPen (deam)	Mbuti	0.256	0.003	74.56	249,291
Hazara	DenPen (all)	Mbuti	0.247	0.004	65.93	106,994	Thai	DenPen (all)	Mbuti	0.235	0.004	60.42	106,502
Hazara	DenPen (deam)	Mbuti	0.249	0.003	74.66	249,282	Thai	DenPen (deam)	Mbuti	0.236	0.003	68.37	247,962
Hezhen	DenPen (all)	Mbuti	0.237	0.004	61.1	106,099	Tlingit	DenPen (all)	Mbuti	0.259	0.004	61.57	101,860
Hezhen	DenPen (deam)	Mbuti	0.238	0.003	68.12	247,196	Tlingit	DenPen (deam)	Mbuti	0.261	0.004	68.47	237,211
Hungarian	DenPen (all)	Mbuti	0.248	0.004	65.69	107,297	Tu	DenPen (all)	Mbuti	0.233	0.004	62.59	106,596
Hungarian	DenPen (deam)	Mbuti	0.252	0.003	74.05	249,865	Tu	DenPen (deam)	Mbuti	0.236	0.003	68.89	248,310
Icelandic	DenPen (all)	Mbuti	0.259	0.004	65.98	107,273	Tubalar	DenPen (all)	Mbuti	0.252	0.004	63.51	106,674
Icelandic	DenPen (deam)	Mbuti	0.262	0.003	76.2	249,628	Tubalar	DenPen (deam)	Mbuti	0.255	0.004	72.69	248,421
Igorot	DenPen (all)	Mbuti	0.236	0.004	58.93	105,342	Tujia	DenPen (all)	Mbuti	0.232	0.004	59.48	106,161
Igorot	DenPen (deam)	Mbuti	0.238	0.004	67.89	245,332	Tujia	DenPen (deam)	Mbuti	0.234	0.003	67.59	247,205
Iranian	DenPen (all)	Mbuti	0.242	0.004	65.01	107,132	Turkish	DenPen (all)	Mbuti	0.24	0.004	65.36	107,256
Iranian	DenPen (deam)	Mbuti	0.244	0.003	75.28	249,539	Turkish	DenPen (deam)	Mbuti	0.244	0.003	73.27	249,942
Iraqi_Jew	DenPen (all)	Mbuti	0.24	0.004	58.32	101,850	Tuscan	DenPen (all)	Mbuti	0.246	0.004	61.18	101,796
Iraqi_Jew	DenPen (deam)	Mbuti	0.242	0.004	66.76	237,113	Tuscan	DenPen (deam)	Mbuti	0.248	0.004	66.59	237,182
Irula	DenPen (all)	Mbuti	0.239	0.004	67.16	106,621	Ulchi	DenPen (all)	Mbuti	0.241	0.004	62.16	105,868
Irula	DenPen (deam)	Mbuti	0.239	0.003	75.46	248,218	Ulchi	DenPen (deam)	Mbuti	0.243	0.004	69.24	246,602
Itelman	DenPen (all)	Mbuti	0.256	0.004	58.61	101,219	Xibo	DenPen (all)	Mbuti	0.232	0.004	60.5	106,362
Itelman	DenPen (deam)	Mbuti	0.258	0.004	65.38	235,743	Xibo	DenPen (deam)	Mbuti	0.234	0.003	68.79	247,866
Japanese	DenPen (all)	Mbuti	0.233	0.004	63.6	108,740	Yadava	DenPen (all)	Mbuti	0.24	0.004	64.57	106,664
Japanese	DenPen (deam)	Mbuti	0.236	0.003	71.36	253,221	Yadava	DenPen (deam)	Mbuti	0.242	0.003	71.27	248,589
Jordanian	DenPen (all)	Mbuti	0.228	0.003	65.42	110,114	Yakut	DenPen (all)	Mbuti	0.245	0.004	64.59	106,062
Jordanian	DenPen (deam)	Mbuti	0.231	0.003	74.39	256,623	Yakut	DenPen (deam)	Mbuti	0.246	0.003	72.47	247,197
Ju_hoan_North	DenPen (all)	Mbuti	0.043	0.002	19.75	109,732	Yemenite_Jew	DenPen (all)	Mbuti	0.227	0.004	61.11	106,953
Ju_hoan_North	DenPen (deam)	Mbuti	0.044	0.002	23.84	254,871	Yemenite_Jew	DenPen (deam)	Mbuti	0.23	0.003	68.27	248,898
Kalash	DenPen (all)	Mbuti	0.254	0.004	65.42	106,142	Yi	DenPen (all)	Mbuti	0.234	0.004	60.38	106,556
Kalash	DenPen (deam)	Mbuti	0.257	0.003	74.22	247,358	Yi	DenPen (deam)	Mbuti	0.235	0.003	67.51	247,904
Kapu	DenPen (all)	Mbuti	0.241	0.004	64.75	106,887	Yoruba	DenPen (all)	Mbuti	0.084	0.003	33.53	108,275
Kapu	DenPen (deam)	Mbuti	0.243	0.003	73.04	248,940	Yoruba	DenPen (deam)	Mbuti	0.083	0.002	38.45	252,124
Karitiana	DenPen (all)	Mbuti	0.268	0.004	65.08	104,685	Zapotec	DenPen (all)	Mbuti	0.268	0.004	66.77	104,872
Karitiana	DenPen (deam)	Mbuti	0.272	0.004	71.62	243,959	Zapotec	DenPen (deam)	Mbuti	0.27	0.004	73.92	244,348

References

1 Lévêque, F. & Miskovsky, J. Le Castelperronien dans son environnement géologique. Essai de synthèse à partir de l'étude lithostratigraphique du remplissage de la grotte de la grande Roche de la Plématrie (Quincay, Vienne) et d'autres dépôts actuellement mis au jour. *Anthropologie (L')(Paris)* **87**, 369-391 (1983).

2 Jacobs, Z., Li, B., Jankowski, N. & Soressi, M. Testing of a single grain OSL chronology across the Middle to Upper Palaeolithic transition at Les Cottés (France). *J Archaeol Sci* **54**, 110-122 (2015).

3 Roussel, M., Soressi, M. & Hublin, J. J. The Chatelperronian conundrum: Blade and bladelet lithic technologies from Quincay, France. *J Hum Evol* **95**, 13-32, doi:10.1016/j.jhevol.2016.02.003 (2016).

4 Welker, F. *et al.* Variations in glutamine deamidation for a Chatelperronian bone assemblage as measured by peptide mass fingerprinting of collagen. *Sci Technol Archaeol* **3**, 15-27, doi:10.1080/20548923.2016.1258825 (2017).

5 Rendu, W. *et al.* Subsistence strategy changes during the Middle to Upper Paleolithic transition reveals specific adaptations of Human Populations to their environment. *Sci Rep* **9**, 1-11, doi:10.1038/s41598-019-50647-6 (2019).

6 Talamo, S., Soressi, M., Roussel, M., Richards, M. & Hublin, J.-J. A radiocarbon chronology for the complete Middle to Upper Palaeolithic transitional sequence of Les Cottés (France). *J Archaeol Sci* **39**, 175-183 (2012).

7 Rigaud, S. *et al.* Les pratiques ornementales à l'Aurignacien ancien dans le Centre-Ouest de la France: l'apport des fouilles récentes aux Cottés (Vienne). *Bulletin de la Société préhistorique française*, 19-38 (2014).

8 Hublin, J.-J. *et al.* Initial Upper Palaeolithic Homo sapiens from Bacho Kiro Cave, Bulgaria. *Nature* **581**, 299-302 (2020).

9 Tsanova, T. *et al.* The Initial Upper Palaeolithic lithic assemblage from Bacho Kiro Cave (Bulgaria). *Paper Presented at the 9th Annual Meeting of the European Society for the Study of Human Evolution, Abstract Book, Virtual Meeting*, 120 (2020).

10 Martisius, N. L. *et al.* Initial Upper Paleolithic bone technology and personal ornaments at Bacho Kiro Cave (Bulgaria). *J Hum Evol* **167**, 103198 (2022).

11 Fewlass, H. *et al.* A (14)C chronology for the Middle to Upper Palaeolithic transition at Bacho Kiro Cave, Bulgaria. *Nat Ecol Evol* **4**, 794-801, doi:10.1038/s41559-020-1136-3 (2020).

12 Reimer, P. J. *et al.* The Intcal20 Northern Hemisphere Radiocarbon Age Calibration Curve (0-55 Cal Kbp). *Radiocarbon* **62**, 725-757, doi:10.1017/Rdc.2020.41 (2020).

13 Ramsey, C. B. Bayesian analysis of radiocarbon dates. *Radiocarbon* **51**, 337-360 (2009).

14 Vasiliev, S. K., Shunkov, M. V. & Kozlikin, M. B. Bone Remains from the Pleistocene Deposits in the South Chamber of Denisova Cave: New Evidence. *Problems of Archaeology, Ethnography, Anthropology of Siberia and Neighboring Territories* **27**, 83-90, doi:10.17746/2658-6193.2021.27.0083-0090 (2021).

15 Agadjanian, A. K., Shunkov, M. V. & Kozlikin, M. B. Taxonomic Composition of Small Vertebrates from the Pleistocene Deposits in the South Chamber of Denisova Cave. *Problems of Archaeology, Ethnography, Anthropology of Siberia and Neighboring Territories* **27**, 7-11, doi:10.17746/2658-6193.2021.27.0007-0011 (2021).

16 Shunkov, M. V., Fedorchenko, A. Y., Kozlikin, M. B. & Derevianko, A. P. Initial Upper Palaeolithic ornaments and formal bone tools from the East Chamber of Denisova Cave in the Russian Altai. *Quatern Int* **559**, 47-67, doi:10.1016/j.quaint.2020.07.027 (2020).
17 Douka, K. *et al.* Age estimates for hominin fossils and the onset of the Upper Palaeolithic at Denisova Cave. *Nature* **565**, 640-644, doi:10.1038/s41586-018-0870-z (2019).
18 Martisius, N. L., McPherron, S. P., Schulz-Kornas, E., Soressi, M. & Steele, T. E. A method for the taphonomic assessment of bone tools using 3D surface texture analysis of bone microtopography. *Archaeol Anthrop Sci* **12**, 1-16, doi:10.1007/s12520-020-01195-y (2020).
19 Martisius, N. L. *et al.* Time wears on: Assessing how bone wears using 3D surface texture analysis. *Plos One* **13**, doi:10.1371/journal.pone.0206078 (2018).
20 Soressi, M. *et al.* Neandertals made the first specialized bone tools in Europe. *Proceedings of the National Academy of Sciences* **110**, 14186-14190 (2013).
21 Rohland, N., Siedel, H. & Hofreiter, M. Nondestructive DNA extraction method for mitochondrial DNA analyses of museum specimens. *Biotechniques* **36**, 814-821, doi:10.2144/04365ST05 (2004).
22 Gomes. Nondestructive extraction DNA method from bones or teeth, true or false? , doi:https://doi.org/10.1016/j.fsigss.2015.09.111 (2015).
23 Schulz, E., Calandra, I. & Kaiser, T. M. Applying Tribology to Teeth of Hoofed Mammals. *Scanning* **32**, 162-182, doi:10.1002/sca.20181 (2010).
24 Schulz-Kornas, E., Kaiser, T. M., Calandra, I. & Winkler, D. E. *A brief history of quantitative wear analyses with an appeal for a holistic view on dental wear processes*. (Verlag Dr. Friedrich Pfeil, 2020).
25 Schulz-Kornas, E. *et al.* Everything matters: Molar microwear texture in goats (Capra aegagrus hircus) fed diets of different abrasiveness. *Palaeogeography, Palaeoclimatology, Palaeoecology* **552**, 109783, doi:10.1016/j.palaeo.2020.109783 (2020).
26 Scott, R. S. *et al.* Dental microwear texture analysis shows within-species diet variability in fossil hominins. *Nature* **436**, 693-695, doi:10.1038/nature03822 (2005).
27 Scott, R. S. *et al.* Dental microwear texture analysis: technical considerations. *J Hum Evol* **51**, 339-349, doi:10.1016/j.jhevol.2006.04.006 (2006).
28 Schulz, E. *et al.* Dietary Abrasiveness Is Associated with Variability of Microwear and Dental Surface Texture in Rabbits. *Plos One* **8**, e56167, doi:10.1371/journal.pone.0056167 (2013).
29 Schulz, E., Calandra, I. & Kaiser, T. M. Feeding ecology and chewing mechanics in hoofed mammals: 3D tribology of enamel wear. *Wear* **300**, 169-179, doi:10.1016/j.wear.2013.01.115 (2013).
30 Evans, A. A. & Donahue, R. E. Laser scanning confocal microscopy: a potential technique for the study of lithic microwear. *J Archaeol Sci* **35**, 2223-2230, doi:10.1016/j.jas.2008.02.006 (2008).
31 Stevens, N. E., Harro, D. R. & Hicklin, A. Practical quantitative lithic use-wear analysis using multiple classifiers. *J Archaeol Sci* **37**, 2671-2678, doi:10.1016/j.jas.2010.06.004 (2010).
32 Evans, A. A. & Macdonald, D. Using Metrology in Early Prehistoric Stone Tool Research: Further Work and a Brief Instrument Comparison. *Scanning* **33**, 294-303, doi:10.1002/sca.20272 (2011).

33 Stemp, W. J. & Chung, S. Discrimination of Surface Wear on Obsidian Tools Using LSCM and RelA: Pilot Study Results (Area-Scale Analysis of Obsidian Tool Surfaces). *Scanning* **33**, 279-293, doi:10.1002/sca.20250 (2011).
34 Macdonald, D. A., Xie, L. Y. & Gallo, T. Here's the dirt: First applications of confocal microscopy for quantifying microwear on experimental ground stone earth working tools. *J Archaeol Sci-Rep* **26**, 101861, doi:10.1016/j.jasrep.2019.05.026 (2019).
35 Stemp, W. J., Morozov, M. & Key, A. J. M. Quantifying lithic microwear with load variation on experimental basalt flakes using LSCM and area-scale fractal complexity (Asfc). *Surf Topogr-Metrol* **3**, 034006, doi:10.1088/2051-672x/3/3/034006 (2015).
36 Rosso, D. E., d'Errico, F. & Queffelec, A. Patterns of change and continuity in ochre use during the late Middle Stone Age of the Horn of Africa: The Porc-Epic Cave record. *Plos One* **12**, e0177298, doi:10.1371/journal.pone.0177298 (2017).
37 d'Errico, F. & Backwell, L. Assessing the function of early hominin bone tools. *J Archaeol Sci* **36**, 1764-1773, doi:10.1016/j.jas.2009.04.005 (2009).
38 Watson, A. S. & Gleason, M. A. A comparative assessment of texture analysis techniques applied to bone tool use-wear. *Surf Topogr-Metrol* **4**, 024002, doi:10.1088/2051-672x/4/2/024002 (2016).
39 Lesnik, J. J. Bone tool texture analysis and the role of termites in the diet of South African hominids. *PaleoAnthropology* **268**, 281 (2011).
40 ISO, B. & STANDARD, B. in *Part 2: Terms, definitions and surface texture parameters (ISO 25178-2: 2012)* (2012).
41 Korlevic, P., Talamo, S. & Meyer, M. A combined method for DNA analysis and radiocarbon dating from a single sample. *Sci Rep* **8**, 4127, doi:10.1038/s41598-018-22472-w (2018).
42 Fewlass, H. *et al.* Pretreatment and gaseous radiocarbon dating of 40-100 mg archaeological bone. *Sci Rep* **9**, 5342, doi:10.1038/s41598-019-41557-8 (2019).
43 Wacker, L., Nemec, M. & Bourquin, J. A revolutionary graphitisation system: Fully automated, compact and simple. *Nucl Instrum Meth B* **268**, 931-934, doi:10.1016/j.nimb.2009.10.067 (2010).
44 Wacker, L. *et al.* Micadas: Routine and High-Precision Radiocarbon Dating. *Radiocarbon* **52**, 252-262, doi:10.1017/S0033822200045288 (2010).
45 Deniro, M. J. & Weiner, S. Chemical, Enzymatic and Spectroscopic Characterization of Collagen and Other Organic Fractions from Prehistoric Bones. *Geochim Cosmochim Ac* **52**, 2197-2206, doi:10.1016/0016-7037(88)90122-6 (1988).
46 Yizhaq, M. *et al.* Quality controlled radiocarbon dating of bones and charcoal from the early Pre-Pottery Neolithic B (PPNB) of Motza (Israel). *Radiocarbon* **47**, 193-206, doi:10.1017/S003382220001969X (2005).
47 D'Elia, M. *et al.* Evaluation of possible contamination sources in the C-14 analysis of bone samples by FTIR spectroscopy. *Radiocarbon* **49**, 201-210, doi:10.1017/S0033822200042120 (2007).
48 Van Klinken, G. J. Bone collagen quality indicators for palaeodietary and radiocarbon measurements. *J Archaeol Sci* **26**, 687-695, doi:10.1006/jasc.1998.0385 (1999).
49 Talamo, S. & Richards, M. A Comparison of Bone Pretreatment Methods for Ams Dating of Samples > 30,000 Bp. *Radiocarbon* **53**, 443-449, doi:10.1017/S0033822200034573 (2011).

50 Talamo, S., Fewlass, H., Maria, R. & Jaouen, K. "Here we go again": the inspection of collagen extraction protocols for C-14 dating and palaeodietary analysis. *Sci Technol Archaeol* **7**, 62-77, doi:10.1080/20548923.2021.1944479 (2021).
51 Slon, V. *et al.* Neandertal and Denisovan DNA from Pleistocene sediments. *Science* **356**, 605-608, doi:10.1126/science.aam9695 (2017).
52 Huson, D. H., Auch, A. F., Qi, J. & Schuster, S. C. MEGAN analysis of metagenomic data. *Genome Res* **17**, 377-386, doi:10.1101/gr.5969107 (2007).
53 Vernot, B. *et al.* Unearthing Neanderthal population history using nuclear and mitochondrial DNA from cave sediments. *Science* **372**, eabf1667, doi:10.1126/science.abf1667 (2021).
54 Li, H. *et al.* The sequence alignment/map format and SAMtools. *bioinformatics* **25**, 2078-2079 (2009).
55 Meyer, M. *et al.* A mitochondrial genome sequence of a hominin from Sima de los Huesos. *Nature* **505**, 403-406, doi:10.1038/nature12788 (2014).
56 Peyregne, S. & Peter, B. M. AuthentiCT: a model of ancient DNA damage to estimate the proportion of present-day DNA contamination. *Genome Biol* **21**, 246, doi:10.1186/s13059-020-02123-y (2020).
57 Weissensteiner, H. *et al.* HaploGrep 2: mitochondrial haplogroup classification in the era of high-throughput sequencing. *Nucleic Acids Res* **44**, W58-63, doi:10.1093/nar/gkw233 (2016).
58 Green, R. E. *et al.* A complete Neandertal mitochondrial genome sequence determined by high-throughput sequencing. *Cell* **134**, 416-426, doi:10.1016/j.cell.2008.06.021 (2008).
59 Bouckaert, R. *et al.* BEAST 2.5: An advanced software platform for Bayesian evolutionary analysis. *PLoS Comput Biol* **15**, e1006650, doi:10.1371/journal.pcbi.1006650 (2019).
60 Katoh, K. & Standley, D. M. MAFFT multiple sequence alignment software version 7: improvements in performance and usability. *Mol Biol Evol* **30**, 772-780, doi:10.1093/molbev/mst010 (2013).
61 Green, R. E. *et al.* A draft sequence of the Neandertal genome. *Science* **328**, 710-722, doi:328/5979/710 10.1126/science.1188021 (2010).
62 Andrews, R. M. *et al.* Reanalysis and revision of the Cambridge reference sequence for human mitochondrial DNA. *Nat Genet* **23**, 147, doi:10.1038/13779 (1999).
63 Kass, R. E. & Raftery, A. E. Bayes Factors. *J Am Stat Assoc* **90**, 773-795, doi:10.1080/01621459.1995.10476572 (1995).
64 Baele, G. *et al.* Improving the Accuracy of Demographic and Molecular Clock Model Comparison While Accommodating Phylogenetic Uncertainty. *Molecular Biology and Evolution* **29**, 2157-2167, doi:10.1093/molbev/mss084 (2012).
65 Leache, A. D., Fujita, M. K., Minin, V. N. & Bouckaert, R. R. Species Delimitation using Genome-Wide SNP Data. *Syst Biol* **63**, 534-542, doi:10.1093/sysbio/syu018 (2014).
66 Tamura, K. & Nei, M. Estimation of the number of nucleotide substitutions in the control region of mitochondrial DNA in humans and chimpanzees. *Mol Biol Evol* **10**, 512-526, doi:10.1093/oxfordjournals.molbev.a040023 (1993).
67 Fu, Q. *et al.* A revised timescale for human evolution based on ancient mitochondrial genomes. *Curr Biol* **23**, 553-559, doi:10.1016/j.cub.2013.02.044 (2013).
68 Benazzi, S. *et al.* The makers of the Protoaurignacian and implications for Neandertal extinction. *Science* **348**, 793-796 (2015).

69 Fu, Q. *et al.* An early modern human from Romania with a recent Neanderthal ancestor. *Nature* **524**, 216-219, doi:10.1038/nature14558 (2015).
70 Fu, Q. *et al.* Genome sequence of a 45,000-year-old modern human from western Siberia. *Nature* **514**, 445-449, doi:10.1038/nature13810 (2014).
71 Fu, Q. *et al.* DNA analysis of an early modern human from Tianyuan Cave, China. *Proceedings of the National Academy of Sciences of the United States of America* **110**, 2223-2227, doi:10.1073/pnas.1221359110 (2013).
72 Krause, J. *et al.* The complete mitochondrial DNA genome of an unknown hominin from southern Siberia. *Nature* **464**, 894-897, doi:10.1038/nature08976 (2010).
73 Ermini, L. *et al.* Complete mitochondrial genome sequence of the Tyrolean Iceman. *Current Biology* **18**, 1687-1693 (2008).
74 Gilbert, M. T. P. *et al.* Paleo-Eskimo mtDNA genome reveals matrilineal discontinuity in Greenland. *Science* **320**, 1787-1789 (2008).
75 Devièse, T. *et al.* Compound-specific radiocarbon dating and mitochondrial DNA analysis of the Pleistocene hominin from Salkhit Mongolia. *Nature communications* **10**, 1-7 (2019).
76 Prüfer, K. *et al.* A genome sequence from a modern human skull over 45,000 years old from Zlatý kůň in Czechia. *Nature ecology & evolution* **5**, 820-825 (2021).
77 Slon, V., Glocke, I., Barkai, R., Gopher, A., Hershkovitz, I., Meyer, M. Mammalian mitochondrial capture, a tool for rapid screening of DNA preservation in faunal and undiagnostic remains, and its application to Middle Pleistocene specimens from Qesem Cave (Israel). *Quatern Int* **398**, 210-218, doi:10.1016/j.quaint.2015.03.039 (2015).
78 Kim, H.-J. *et al.* The first complete mitogenome of Cervus canadensis nannodes (Merriam, 1905). *Mitochondrial DNA Part B* **5**, 2294-2296 (2020).
79 Li, H. & Durbin, R. Fast and accurate short read alignment with Burrows-Wheeler transform. *Bioinformatics* **25**, 1754-1760, doi:10.1093/bioinformatics/btp324 (2009).
80 Meyer, M. *et al.* A high-coverage genome sequence from an archaic Denisovan individual. *Science* **338**, 222-226, doi:10.1126/science.1224344 (2012).
81 Meiri, M. *et al.* Faunal record identifies Bering isthmus conditions as constraint to end-Pleistocene migration to the New World. *Proceedings of the Royal Society B: Biological Sciences* **281**, 20132167 (2014).
82 Polziehn, R. O. & Strobeck, C. A phylogenetic comparison of red deer and wapiti using mitochondrial DNA. *Molecular Phylogenetics and Evolution* **22**, 342-356 (2002).
83 Meiri, M. *et al.* Subspecies dynamics in space and time: A study of the red deer complex using ancient and modern DNA and morphology. *J Biogeogr* **45**, 367-380, doi:10.1111/jbi.13124 (2018).
84 Wada, K., Nishibori, M. & Yokohama, M. The complete nucleotide sequence of mitochondrial genome in the Japanese Sika deer (Cervus nippon), and a phylogenetic analysis between Cervidae and Bovidae. *Small ruminant research* **69**, 46-54 (2007).
85 Darriba, D., Taboada, G. L., Doallo, R. & Posada, D. jModelTest 2: more models, new heuristics and parallel computing. *Nat Methods* **9**, 772, doi:10.1038/nmeth.2109 (2012).
86 Wood, D. E. & Salzberg, S. L. Kraken: ultrafast metagenomic sequence classification using exact alignments. *Genome Biol* **15**, R46, doi:10.1186/gb-2014-15-3-r46 (2014).
87 Patterson, N. *et al.* Ancient admixture in human history. *Genetics* **192**, 1065-1093, doi:10.1534/genetics.112.145037 (2012).

88 Patterson, N., Price, A. L. & Reich, D. Population structure and eigenanalysis. *PLoS Genet* **2**, e190, doi:10.1371/journal.pgen.0020190 (2006).
89 Price, A. L. *et al.* Principal components analysis corrects for stratification in genome-wide association studies. *Nat Genet* **38**, 904-909, doi:10.1038/ng1847 (2006).
90 Petr, M., Vernot, B. & Kelso, J. admixr—R package for reproducible analyses using ADMIXTOOLS. *Bioinformatics* **35**, 3194-3195 (2019).
91 Mallick, S. *et al.* The Simons Genome Diversity Project: 300 genomes from 142 diverse populations. *Nature* **538**, 201-206, doi:10.1038/nature18964 (2016).
92 Moreno-Mayar, J. V. *et al.* Early human dispersals within the Americas. *Science* **362**, eaav2621 (2018).
93 Narasimhan, V. M. *et al.* The formation of human populations in South and Central Asia. *Science* **365**, eaat7487 (2019).
94 Posth, C. *et al.* Reconstructing the deep population history of Central and South America. *Cell* **175**, 1185-1197. e1122 (2018).
95 Yu, H. *et al.* Paleolithic to Bronze Age Siberians reveal connections with first Americans and across Eurasia. *Cell* **181**, 1232-1245. e1220 (2020).
96 Meyer, M. *et al.* Nuclear DNA sequences from the Middle Pleistocene Sima de los Huesos hominins. *Nature* **531**, 504-507, doi:10.1038/nature17405 (2016).
97 Hajdinjak, M. *et al.* Reconstructing the genetic history of late Neanderthals. *Nature* **555**, 652-656, doi:10.1038/nature26151 (2018).
98 R: A Language and Environment for Statistical Computing (R Foundation for Statistical Computing, http://www.R-project.org/ 2013).
99 Damgaard, P. *et al.* The first horse herders and the impact of early Bronze Age steppe expansions into Asia. *Science* **360**, eaar7711 (2018).
100 Sikora, M. *et al.* The population history of northeastern Siberia since the Pleistocene. *Nature* **570**, 182-188 (2019).
101 Fu, Q. *et al.* The genetic history of Ice Age Europe. *Nature* **534**, 200-205, doi:10.1038/nature17993 (2016).
102 Raghavan, M. *et al.* Upper Palaeolithic Siberian genome reveals dual ancestry of Native Americans. *Nature* **505**, 87-91, doi:10.1038/nature12736 (2014).
103 Sikora, M. *et al.* Ancient genomes show social and reproductive behavior of early Upper Paleolithic foragers. *Science* **358**, 659-662 (2017).
104 Massilani, D. *et al.* Denisovan ancestry and population history of early East Asians. *Science* **370**, 579-583 (2020).
105 Jones, E. R. *et al.* Upper Palaeolithic genomes reveal deep roots of modern Eurasians. *Nature communications* **6**, 1-8 (2015).
106 Busing, F. M., Meijer, E. & Leeden, R. V. D. Delete-m jackknife for unequal m. *Statistics and Computing* **9**, 3-8 (1999).
