# Supplementary material for: Ancient human DNA recovered from a Palaeolithic pendant
Source: Nature. 2023 May 3;618(7964):328–32. doi: 10.1038/s41586-023-06035-2 (PMC10247382; doi:10.1038/s41586-023-06035-2)
Supplement: Supplementary file 2 — Reporting Summary [file 41586_2023_6035_MOESM2_ESM.pdf]

nature portfolio

Corresponding author(s): Elena Essel, Marie Soressi, Matthias Meyer

Last updated by author(s): Mar 15, 2023

Reporting Summary

Nature Portfolio wishes to improve the reproducibility of the work that we publish. This form provides structure for consistency and transparency in reporting. For further information on Nature Portfolio policies, see our Editorial Policies and the Editorial Policy Checklist.

Statistics

For all statistical analyses, confirm that the following items are present in the figure legend, table legend, main text, or Methods section.

n/a	Confirmed	
☐	☒	The exact sample size (*n*) for each experimental group/condition, given as a discrete number and unit of measurement
☐	☒	A statement on whether measurements were taken from distinct samples or whether the same sample was measured repeatedly
☐	☒	The statistical test(s) used AND whether they are one- or two-sided<br>*Only common tests should be described solely by name; describe more complex techniques in the Methods section.*
☒	☐	A description of all covariates tested
☒	☐	A description of any assumptions or corrections, such as tests of normality and adjustment for multiple comparisons
☐	☒	A full description of the statistical parameters including central tendency (e.g. means) or other basic estimates (e.g. regression coefficient) AND variation (e.g. standard deviation) or associated estimates of uncertainty (e.g. confidence intervals)
☐	☒	For null hypothesis testing, the test statistic (e.g. *F*, *t*, *r*) with confidence intervals, effect sizes, degrees of freedom and *P* value noted<br>*Give P values as exact values whenever suitable.*
☐	☒	For Bayesian analysis, information on the choice of priors and Markov chain Monte Carlo settings
☒	☐	For hierarchical and complex designs, identification of the appropriate level for tests and full reporting of outcomes
☒	☐	Estimates of effect sizes (e.g. Cohen's *d*, Pearson's *r*), indicating how they were calculated

Our web collection on statistics for biologists contains articles on many of the points above.

Software and code

Policy information about availability of computer code

Data collection	No software was used for the collection of data.
Data analysis	All software packages used for analysis are publicly available and cited in the Online Methods section or in the Supplementary Information.<br>These include together with their applications:<br>BAM file handling: samtools (version 1.3.1),<br>Metagenomics analysis: BLAST and MEGAN (version 0.0.12, see Slon et al., 2017),<br>f3- and D-statistics: ADMIXTOOLS (version 5.1) and R package admixr (version 0.7.1),<br>Adapter trimming and overlap-merging of paired-end reads: leeHom (https://github.com/mpieva/leeHom/tree/v.1.1.5),<br>Contamination estimates: AuthentiCT (https://github.com/StephanePeyregne/AutheticT, version 1.0.0),<br>Haplogroup assignment: Haplogrep2 (version2.4.0),<br>Tree buiding and genetic dating: BEAST2 (version 2.6.6),<br>PCA: smartpca from EIGENSOFT package (version 8.0.0),<br>Radiocarbon date calibration: IntCal2020 and OxCal platform (version 4.4),<br>3DST analysis: Mountains Map® Premium, version 7.4.8076 (Digital Surf (Besançon, France)),<br>FTIR analysis: Resolution Pro software (Agilent Technologies, version 5.3.0.1964),<br>mtDNA sequence alignment: MAFFT (version 7.453),<br>Clock and tree model selection: BEAST2's MODEL_SELECTION package,<br>Combination of log and tree files: BEAST2's logcombiner2,<br>Tree annotation: BEAST2's treeannotator program,<br>Tree visualization: Figtree (v1.4.4, https://github.com/rambaut/figtree/),<br>Tree tip dating: BEAST2's Tracer program,

Sequence mapping: Burrows-Wheeler Aligner (BWA, version 0.5.10-evan.9-1-g44db244),
PCR duplicate removal: bam-rmdup (https://github.com/mpieva/biohazard-tools/tree/v0.2-knowngood),
Nucleotide substitution model selection: jModelTest (version 2.1),
Identification of primate sequences: Kraken (version 1),
Genotype calling: bam-caller version 0.2 (https://github.com/bodkan/bam-caller, 369)

For manuscripts utilizing custom algorithms or software that are central to the research but not yet described in published literature, software must be made available to editors and reviewers. We strongly encourage code deposition in a community repository (e.g. GitHub). See the Nature Portfolio guidelines for submitting code & software for further information.

Data

Policy information about availability of data

All manuscripts must include a data availability statement. This statement should provide the following information, where applicable:

- Accession codes, unique identifiers, or web links for publicly available datasets
- A description of any restrictions on data availability
- For clinical datasets or third party data, please ensure that the statement adheres to our policy

Supplementary Information is available for this paper.
Supplementary Data file 1. Overview of DNA lysates, extracts and libraries prepared in this study and results of the mammalian and human mtDNA captures.
Supplementary Data file 2. Overview and summary statistics of the human nuclear DNA captures targeting 470,724 positions in the genome.

Cervid mtDNA sequences used for probe design, mapping and phylogenetic reconstructions were retrieved from NCBI GenBank, under accession numbers AB245427, JN632610, NC_020700, NC_050863, MG020563-MG020567 and MG020569-MG020571.

The reconstructed ancient human and cervid mtDNA sequences can be found on Dryad at https://doi.org/10.5061/dryad.41ns1rnj1 along with the SNPs used in the nuclear DNA enrichment. Sequencing data have been deposited in the ENA under project number PRJEB56213.

Human research participants

Policy information about studies involving human research participants and Sex and Gender in Research.

Reporting on sex and gender	N/A
Population characteristics	N/A
Recruitment	N/A
Ethics oversight	N/A

Note that full information on the approval of the study protocol must also be provided in the manuscript.

Field-specific reporting

Please select the one below that is the best fit for your research. If you are not sure, read the appropriate sections before making your selection.

☒ Life sciences ☐ Behavioural & social sciences ☐ Ecological, evolutionary & environmental sciences

For a reference copy of the document with all sections, see nature.com/documents/nr-reporting-summary-flat.pdf

Life sciences study design

All studies must disclose on these points even when the disclosure is negative.

Sample size	Sample sizes were limited by the availability of suitable archaeological material for potentially destructive analyses. For testing DNA extraction reagents, two independent samples were used per reagent, and four measurements obtained per sample. The statistical power achieved in these experiments is reflected in the significance values. Only four cleanly excavated objects could be obtained for non-destructive DNA extraction. These objects were analyzed and characterized independently of each other; no data was combined for statistical testing.
Data exclusions	No samples were excluded from this study.
Replication	Technical experiments were replicated using two samples per condition. DNA library preparation was replicated for the two DNA extracts from DCP1 that yielded the highest number of ancient human DNA sequences in non-destructive DNA extraction (five libraries each extract, see Data file S1).
Randomization	Randomisation was not relevant to this study because the analysis of DNA sequence data performed here does not involve procedures that depend on subjective evaluation or are otherwise dependent on preconceived hypotheses. In addition, randomization and blinding are

precluded by the unique shape of the objects analyzed.

Blinding	All data come from archaeological objects that are unique in size and shape, making blinding impossible.

Reporting for specific materials, systems and methods

We require information from authors about some types of materials, experimental systems and methods used in many studies. Here, indicate whether each material, system or method listed is relevant to your study. If you are not sure if a list item applies to your research, read the appropriate section before selecting a response.

Materials & experimental systems

n/a	Involved in the study
☒	☐ Antibodies
☒	☐ Eukaryotic cell lines
☐	☒ Palaeontology and archaeology
☒	☐ Animals and other organisms
☒	☐ Clinical data
☒	☐ Dual use research of concern

Methods

n/a	Involved in the study
☒	☐ ChIP-seq
☒	☐ Flow cytometry
☒	☐ MRI-based neuroimaging

Palaeontology and Archaeology

Specimen provenance	All permits for excavation at Denisova Cave were obtained by the Institute of Archaeology and Ethnography, Siberian Branch of the Russian Academy of Science from the Ministry of Culture of the Russian Federation, and molecular analyses of the material granted as part of an agreement of scientific cooperation between the Institute of Archaeology and Ethnography, Siberian Branch of the Russian Academy of Sciences and the Max Planck Institute for Evolutionary Anthropology for projects in the field of palaeogenetics in North Asia, signed on December 25, 2018 and valid for a duration of five years. Permits for excavations and molecular analyses at Bacho Kiro were provided by the Bulgarian Ministry of Culture and the National Museum of Natural History, Sofia, Bulgaria (permit for regular archaeological investigation №RAP-120-11, signed on May 5, 2019). Permits for excavation at Les Cottés and Quincay were provided by the French Ministry of Culture. Approval for molecular analysis was granted through agreements of cooperation between the Max Planck Institute for Evolutionary Anthropology and M. Soressi representing the owners of the Les Cottés and Quincay collections.
Specimen deposition	Specimens used in this study are currently stored at the Max Planck Institute for Evolutionary Anthropology and will be returned to the owning institutions after publication of the study.
Dating methods	Two new radiocarbon dates were obtained from charcoal samples from layer 11 in the South chamber of Denisova Cave. Pre-treatment was done with the AOx-SC method, pre-treatment and dating were performed at the Oxford Radiocarbon Accelerator Unit in Oxford, UK. All previously published radiocarbon dates were newly re-calibrated using IntCal20 (see Supplementary information).

☒ Tick this box to confirm that the raw and calibrated dates are available in the paper or in Supplementary Information.

Ethics oversight	Ethical and legal concerns associated with excavation, material transfer and molecular analyses were addressed as part of the permits and scientific cooperation agreements cited above (see 'Specimen provenance').

Note that full information on the approval of the study protocol must also be provided in the manuscript.
